# Plasma proteome profiling identifies XPNPEP3 as a novel biomarker associated with metabolic dysfunction-associated steatotic liver disease in patients with type 2 diabetes mellitus

**DOI:** 10.1080/07853890.2026.2654911

**Published:** 2026-04-13

**Authors:** Hua Ji, Yating Lu, Yichang Liu, Yinguang Jia, Jie Liu, Datong Deng, Mingwei Chen

**Affiliations:** a Department of Endocrinology, The First Affiliated Hospital of Anhui Medical University, Anhui Medical University, Hefei City, Anhui Province, China; b Department of General Practice, The First Affiliated Hospital of Anhui Medical University, Anhui Medical University, Hefei City, Anhui Province, China; c Department of Pathology, The First Affiliated Hospital of Anhui Medical University, Anhui Medical University, Hefei City, Anhui Province, China; d Pathology Center of Anhui Medical University, Hefei City, Anhui Province, China; e Institute of Endocrinology and Metabolism, Anhui Medical University, Hefei City, Anhui Province, China

**Keywords:** Type 2 diabetes mellitus, metabolic dysfunction-associated steatotic liver disease, plasma proteomics, XPNPEP3, biomarkers

## Abstract

**Objective:**

To identify plasma protein differences between type 2 diabetes mellitus (T2DM) patients with and without metabolic dysfunction-associated steatotic liver disease (MASLD), and to evaluate the diagnostic potential of X-prolyl aminopeptidase 3 (XPNPEP3) for identifying MASLD in T2DM patients.

**Methods:**

Twenty T2DM inpatients were categorized into groups with and without MASLD and their plasma samples were analyzed using data-independent acquisition mass spectrometry, followed by bioinformatics analysis to identify differentially expressed proteins. The cohort was then expanded to 84 patients, and plasma XPNPEP3 levels were validated by enzyme-linked immunosorbent assay. Correlation between XPNPEP3 and clinical indicators were evaluated, and diagnostic performance was determined via receiver operating characteristic (ROC) analysis. Immunohistochemistry was employed to compare hepatic XPNPEP3 expression between the two groups.

**Results:**

Proteomic analysis identified 176 differentially expressed proteins, with XPNPEP3 exhibiting the most significant down-regulation by fold change. In the validation cohort, plasma XPNPEP3 was significantly lower in T2DM+MASLD versus T2DM alone. XPNPEP3 levels were negatively correlated with diabetes duration, liver function markers, and triglyceride levels, and was identified as an independent factor inversely associated with MASLD in T2DM.ROC analysis demonstrated strong diagnostic performance for XPNPEP3, further enhanced when combined with BMI and diabetes duration.  Immunohistochemistry confirmed reduced hepatic XPNPEP3 expression in T2DM+MASLD patients.

**Conclusions:**

Lower plasma XPNPEP3 is independently associated with MASLD in T2DM patients and demonstrates strong diagnostic potential, positioning XPNPEP3 as a promising biomarker for diagnosing MASLD in T2DM patients and a novel target for non-invasive diagnostic tool development.

## KEY MESSAGES

1. Plasma proteomic profiling identifies XPNPEP3 as a novel and significantly downregulated biomarker in patients with T2DM and comorbid MASLD.
2. Plasma XPNPEP3 levels are independently and inversely associated with MASLD in T2DM and show good discriminatory ability, particularly when combined with clinical indicators such as BMI and diabetes duration.
3. The downregulation of XPNPEP3, confirmed at the target organ level in liver tissue, underscores its role in the pathophysiology linking T2DM and MASLD.

## Introduction

Metabolic dysfunction-associated steatotic liver disease (MASLD), previously referred to as non-alcoholic fatty liver disease (NAFLD), is a chronic liver condition linked to metabolic dysfunction and characterized by abnormal fat accumulation in the liver. The disease spectrum includes simple steatosis, metabolic-associated steatohepatitis (MASH), liver fibrosis, cirrhosis, and eventually hepatocellular carcinoma (HCC) [1]. The risk of progressing to HCC increases markedly with disease stage. While the annual incidence among patients with non-cirrhotic MASH is low (approximately 0.01%-0.13%), it rises substantially to 0.5%-2.6% in those who develop MASH-related cirrhosis [2]. The differentiation between these stages, particularly the progression from simple steatosis to MASH and the grading of fibrosis, is primarily based on histopathological assessment of liver biopsy, which remains the diagnostic gold standard. In addition, non-invasive tools such as serum biomarkers (e.g. FIB-4) and elastography (e.g. FibroScan) are increasingly used for risk stratification and monitoring. HCC is typically diagnosed through imaging modalities and/or biopsy following established clinical guidelines [3].

MASLD is one of the leading causes of chronic liver disease globally, affecting 38% of the world’s population [4]. In the Asia-Pacific region, the epidemiological burden is especially high, with the prevalence of MASLD ranging from 28% to 40% according to the 2025 Asia-Pacific Association for the Study of Liver (APASL) clinical practice guidelines [5]. By 2030, China is projected to have 314.58 million patients with MASLD, making it the fastest-growing country in terms of the disease burden [6]. Concurrently, the global incidence and prevalence of type 2 diabetes mellitus (T2DM) have risen significantly, now affecting one in ten individuals aged 20 to 79 [7].

The high prevalence of MASLD is closely associated with the rise in T2DM, and the two conditions frequently coexist, posing a serious threat to public health. The incidence of MASLD in T2DM patients (65%-70%) is more than twice that of the general population (20%-30%) [8]. Furthermore, T2DM has been identified as an independent risk factor for liver decompensation and HCC in MASLD patients [5]. Both diseases share similar pathophysiological mechanisms, with insulin resistance (IR) being a key pathogenic factor. T2DM promotes the development of MASLD by disrupting glucose and lipid metabolism through IR, chronic inflammation, and oxidative stress [9]. Conversely, MASLD exacerbates the progression of T2DM and its complications through the same pathogenic processes [10]. This reciprocal interaction heightens the risk of both hepatic and extrahepatic adverse outcomes [11]. A central factor in this cycle is mitochondrial dysfunction, which plays a pivotal role in the pathogenesis of both T2DM and MASLD [12]. Liver mitochondria, as key regulators of energy metabolism, lipid homeostasis, and oxidative balance, are crucial for nutrient oxidation and energy production [13]. Under IR conditions, excessive free fatty acid delivery to the liver impairs mitochondrial β-oxidation capacity, leading to lipid accumulation, increased reactive oxygen species (ROS) production, and oxidative stress [14]. This cascade further aggravates the progression of MASLD.

Ultrasound is currently the primary diagnostic tool for hepatic steatosis, though its accuracy significantly decreases in patients with mild steatosis (<20% fat infiltration) or severe obesity (body mass index [BMI] > 40 kg/m^2^) [1]. While liver biopsy offers a definitive diagnosis, its invasive nature limits its clinical applicability. Thus, non-invasive diagnostic methods for hepatic steatosis are critical for effective MASLD diagnosis [15]. The plasma proteome, reflecting the body’s overall physiological and pathological state, is a valuable resource for investigating underlying mechanisms and identifying potential biomarkers [16]. Recent advancements in data-independent acquisition (DIA) mass spectrometry, particularly the directDIA method, have superseded traditional data-dependent acquisition (DDA) technology [17]. This approach enables comprehensive peptide profiling by continuously fragmenting all peptide precursor ions at designated time intervals [18], combining the high reproducibility and wide dynamic range of DIA with the user-friendly attributes of DDA [19]. Despite studies exploring proteomic changes in T2DM [20–21] or MASLD [22–24] individually, the distinct proteomic features of T2DM comorbid with MASLD remain less characterized. Uncovering these features is crucial for understanding disease onset and progression, as well as identifying novel diagnostic and therapeutic targets.

In our preliminary proteomic screen, X-prolyl aminopeptidase 3 (XPNPEP3) was selected as the top candidate for validation because it demonstrated both the greatest magnitude of downregulation (largest fold change) and the highest statistical significance (smallest *P*-value). XPNPEP3 is a mitochondrial aminopeptidase that cleaves peptides at the N-terminus of proline residues, thereby participating in the processing and stability regulation of mitochondrial proteins [25]. Its deficiency can lead to markedly reduced assembly and activity of respiratory chain complex I, directly impairing oxidative phosphorylation capacity [26]. This is accompanied by a decline in mitochondrial oxygen consumption rate and a reduction in the abundance of ATP synthase complexes [27]. Thus, by regulating mitochondrial protein processing, respiratory chain function, and ROS metabolism, XPNPEP3 serves as a key molecule in maintaining mitochondrial homeostasis and energy production. Abnormal XPNPEP3 expression has been linked to several diseases: biallelic mutations in XPNPEP3 can cause mitochondrial syndromes, which include renal tuberculosis, exercise intolerance, rhabdomyolysis, and complex neurological symptoms [28–29]. In the cardiovascular system, XPNPEP3 in platelets serves as a potential diagnostic marker for acute myocardial infarction [30] and plays a role in cardiomyopathy by maintaining mitochondrial protein balance [31]. In cancer, it is a transcriptional target of the Wnt/β-catenin signaling pathway, promoting colorectal cancer progression [32], and functions as a prognostic marker for esophageal squamous cell carcinoma [33]. Additionally, splice variants of XPNPEP3 in brain tissue are linked to Alzheimer’s disease susceptibility [34]. However, its role in metabolic diseases, particularly in T2DM with MASLD, remains unexplored. Given its established connection to mitochondrial function, which is critical to both conditions, XPNPEP3 is a protein deserving further investigation.

This study utilized directDIA mass spectrometry to comprehensively analyze plasma proteomic differences between T2DM patients with and without MASLD. Bioinformatics analysis was conducted to identify key differential plasma proteins and dysregulated biological pathways, enabling the identification of specific biomarkers related to the disease and their potential for diagnosing MASLD in the context of T2DM.

## Materials and methods

### Study design and participants

All participants were consecutively recruited from patients with T2DM hospitalized in the Department of Endocrinology at the First Affiliated Hospital of Anhui Medical University between January 2025 and September 2025. Among the screened patients, 135 patients met the initial inclusion criteria for T2DM diagnosis and did not meet any of the exclusion criteria. Of these, 66 were T2DM patients without MASLD, and 69 were T2DM patients with MASLD. The T2DM diagnosis adhered to the American Diabetes Association (ADA) criteria [35], while MASLD was diagnosed according to the Asia-Pacific Association for the Study of the Liver (APASL) guidelines [5]. Exclusion criteria were as follows: other liver diseases (e.g. autoimmune liver diseases, alcohol-related liver diseases, viral hepatitis); any malignancy; severe dysfunction of the kidneys, liver, or heart; acute conditions (e.g. fever, acute infection, acute myocardial infarction); other endocrine disorders (e.g. hyperthyroidism, hypothyroidism); use of fat-generating drugs or fatty liver-modifying medications [e.g. Insulin-sensitizing agents, GLP-1 receptor agonists, SGLT2 inhibitors, lipid-lowering agents (statins), weight-loss medications] in the past six months; and heavy smokers. All patients received diet therapy as the foundation of their T2DM management. They were permitted to continue their routine antidiabetic medications not listed in the exclusion criteria (e.g. sulfonylureas, α-glucosidase inhibitors, insulin) during the study period, as our aim was to reflect real-world clinical practice in the T2DM population.

**Figure 1.**
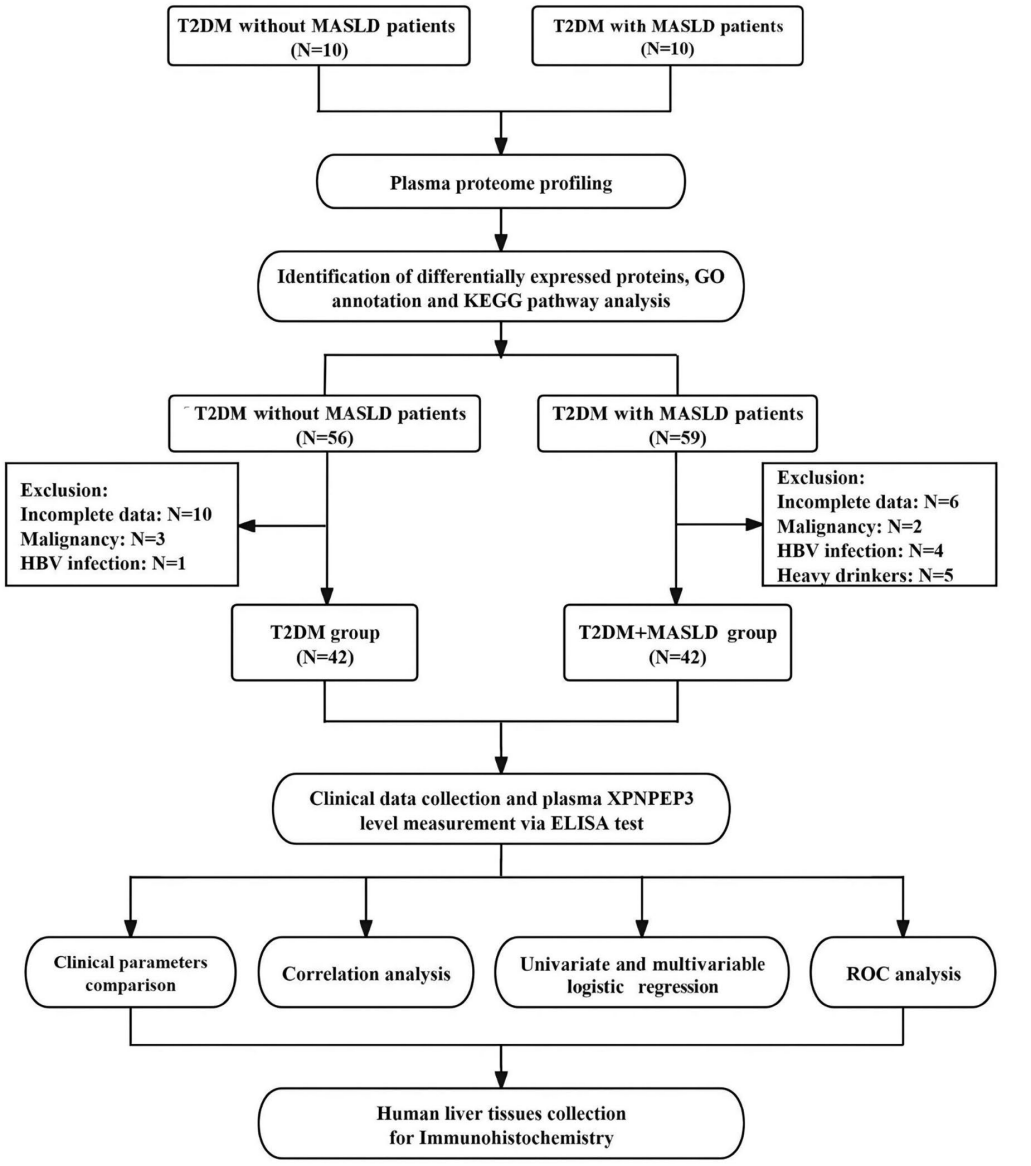
Study design flowchart. T2DM: Type 2 Diabetes Mellitus; MASLD: Metabolic Dysfunction-associated Steatotic Liver Disease; GO: Gene Ontology; KEGG: Kyoto Encyclopedia of Genes and Genomes; HBV: Hepatitis B Virus; ROC: Receiver Operating Characteristic.

After further application of the exclusion criteria and removal of participants with missing clinical data or unsuitable plasma samples, 31 patients were excluded, resulting in a final cohort of 104 participants. The experimental groups were as follows: i) For the discovery phase, 10 newly diagnosed T2DM patients without MASLD and 10 newly diagnosed T2DM patients with MASLD were selected; ii) For the validation phase, 42 patients were included in the T2DM group and 42 in the T2DM + MASLD group. Figure 1 illustrates the research procedure flowchart. All procedures involving human participants adhered to the ethical standards outlined in the 1964 Declaration of Helsinki and its subsequent amendments. This study received approval from the Medical Ethics Committee of the First Affiliated Hospital of Anhui Medical University (approval no. PJ 2025-09-38), and written informed consent was obtained from all participants.

### Sample size consideration and power analysis

Based on the study design described above, the sample sizes for the discovery and validation phases were determined as follows. For the discovery-phase proteomic screening, a sample size of 10 participants per group was selected based on feasibility and was consistent with the scale of preliminary, hypothesis-generating proteomic studies [22,36]. For the independent validation cohort, a prospective sample size calculation was performed. Based on the range of effect sizes (Cohen’s d ranging from approximately 0.6–1.47) observed in prior similar studies [37–39], a conservative target effect size of *d* = 0.7 was therefore set. To detect this effect with 80% power at a two-sided α of 0.05, the minimum required sample size was calculated to be 32 participants per group. Our final validation cohort of 42 participants per group exceeded this requirement, ensuring robust statistical power.

### Plasma sample collection and preparation

After an overnight fast of over 12 h, a 5 mL blood sample was collected from the antecubital vein of each subject into an anticoagulant collection tube the following morning. The blood was then centrifuged at 3000 rpm for 10 min at 4 °C. The supernatant was carefully removed and mixed with protease inhibitors. The plasma was fractionated and stored at −80 °C until further analysis.

For plasma protein spectrum detection before sample preparation, protein enrichment was first conducted. A 20 μL suspension of magnetic particles was placed on a magnetic stand for 2 min to separate and discard the supernatant by magnetic separation. The magnetic particles were then washed five times with a magnetic particle cleaning solution (1300 rpm), and resuspended. To this, 100 μL of plasma was added and incubated for 1 h at 37 °C with shaking at 1000 rpm. After magnetic separation, the supernatant was discarded, and the enriched low-abundance proteins were obtained by washing three times with the magnetic microparticle cleaning solution. Next, proteolytic hydrolysis was performed. A reducing alkylating agent (40 μL) was added to the cleaned magnetic beads and incubated for 5 min at 95 °C with shaking at 1000 rpm. After returning to room temperature, 15 μL of trypsin was added, and digestion was carried out at 37 °C for 2 h at 1000 rpm. The reaction was terminated by adding 55 μL of termination reagent. Peptide desalting was then performed using a desalting column (centrifuged at 700 g for 1 min). The column was washed twice with peptide cleaning reagent and eluted twice with peptide eluent reagent. The eluate was concentrated using a vacuum freeze centrifuge and redissolved in mobile phase A (0.1% formic acid aqueous solution) for subsequent analysis.

### Nano liquid chromatography-tandem mass spectrometry analysis

For each sample, 500 ng of total peptides were separated and analyzed using a nano-UPLC (Vanquish neo) coupled to an Astral instrument (Thermo Scientific) with a nano-electrospray ion source. Separation was achieved with a reversed-phase column (EASY-Spray^™^ HPLC, 150 μm × 15 cm, Thermo Scientific, USA). The mobile phases consisted of phase A (H2O with 0.1% formic acid) and phase B (80% acetonitrile with 0.1% formic acid). A 6.9-minute gradient was used for sample separation.

DIA was performed in profile and positive mode with an Orbitrap analyzer at a resolution of 240K and an m/z range of 380–980 for MS1, and 150–2000 for MS2. High-energy collision dissociation was applied with a normalized collision energy (NCE) of 25% and an isolation window of 2 m/z.

### Spectronaut database search

Vendor raw MS files were processed using DIA-NN software (1.8.1). MS spectra were searched against species-level UniProt FASTA databases (uniprot_Homosapiens_9606_ reviewed_2025_05.fasta), with carbamidomethyl [C] as a fixed modification and oxidation (M) and acetyl (protein N-term) as variable modifications. Trypsin was used as the protease, with a maximum of two missed cleavages allowed. The false discovery rate (FDR) was set at 0.01 for both PSM and peptide levels. Peptide identification was performed with an initial precursor mass deviation of up to 20 ppm and a fragment mass deviation of 20 ppm, while all other parameters were retained as default.

### Bioinformatics analysis

Proteins were filtered based on the number of unique peptides, retaining only those with ≥ 2 unique peptide. Missing values from the raw data were imputed using the minimum one-half method. After preprocessing, a total of 3152 proteins were retained. Data were logged and centralized using the stats package in R software (version 3.6.3), followed by principal component analysis (PCA) to assess clustering trends and overall distribution. Statistical methods were applied to identify differentially expressed proteins (DEPs), with the screening criteria for DEPs being a *P*-value < 0.05 from Student’s t-test or chi-square test and a fold change ≤ 0.83 or ≥ 1.2. This fold change threshold is commonly used for initial screening in exploratory proteomics studies to efficiently enrich for proteins with appreciable abundance changes [40–42]. Hierarchical clustering analysis of DEPs was conducted and visualized as heat maps using the pheatmap package in R software to explore the differences in DEPs between the two groups. Enrichment analysis of DEPs was performed using the GO database (http://ftp.ebi.ac.uk/pub/databases/GO/ goa/proteomes), which provided an analysis of the Gene Ontology (GO) enrichment for biological processes, cellular localization, and molecular functions. DEPs-enriched pathways were assessed through Kyoto Encyclopedia of Genes and Genomes (KEGG) pathway analysis (http://rest.kegg.jp), with *p* < 0.05 considered statistically significant. Protein-protein interaction (PPI) networks of DEPs were constructed using the STRING database (http://string-db.org).

### General information and laboratory parameters collection

Trained staff collected clinical data from all participants, including age, sex, BMI, duration of diabetes, hypertension history, and medication use within the past 6 months. Blood samples were drawn from the antecubital veins the following morning after participants fasted for at least 12 h. Venous blood samples were analyzed for various laboratory parameters, including fasting plasma glucose (FPG), glycosylated hemoglobin A1c (HbA1c), hemoglobin (Hb), liver function indicators (albumin [ALB], alanine aminotransferase [ALT], aspartate aminotransferase [AST], alkaline phosphatase [ALP], gamma-glutamyl transpeptidase [GGT]), renal function markers (serum creatinine [Cre]), and lipid components (triglyceride [TG], total cholesterol [TCH], low-density lipoprotein cholesterol [LDL-C], high-density lipoprotein cholesterol [HDL-C]).

### Measurement of plasma XPNPEP3 levels

Plasma levels of XPNPEP3 were measured using enzyme-linked immunosorbent assay (ELISA). The kit was sourced from Jiangsu Jingmei Biotechnology Co., Ltd. (cat. no. J0506-HA). It has a sensitivity of 0.25 pg/mL, with intra- and inter-assay coefficients of variation of 5.4% and 7.6%, respectively. The XPNPEP3 expression level in plasma samples was determined by measuring absorbance at 450 nm using a microplate reader (Labsystems Multiskan M-S). The assay procedure followed the manufacturer’s instructions precisely.

### Immunohistochemical (IHC) staining of liver tissues

To validate XPNPEP3 expression at the tissue level, paraffin-embedded liver tissue samples were collected from 24 T2DM patients (12 in the T2DM group and 12 in the T2DM + MASLD group) from the Department of Pathology at the hospital. The basic clinical characteristics of these patients are provided in Table SI. The experiment was approved by the Medical Ethics Committee of the First Affiliated Hospital of Anhui Medical University, and informed consent was obtained from all subjects. Tissue sections were cut at 5 μm thickness, deparaffinized, and rehydrated. Antigen retrieval was performed using EDTA buffer (pH 8.0) in a pressure cooker. Endogenous peroxidase activity was blocked with 3% hydrogen peroxide, followed by incubation with anti-XPNPEP3 primary antibody (1:150; cat. no. BD-PE4472; Suzhou Boaolong Technology Co., Ltd.) at 37 °C for 60 min. HRP-conjugated secondary antibody (cat. no. PV6000; Beijing Zhongshan Jinqiao Biotechnology Co., Ltd.) was then applied at 37 °C for 20 min. Staining was visualized using DAB chromogen, and sections were counterstained with hematoxylin. Images were captured using a light microscope. For quantitative analysis, five randomly selected fields per section were captured at ×400 magnification. The average optical density (AOD) was calculated using ImageJ software (AOD = integrated optical density/area of the selected tissue region) for each field, and the mean value of the five fields was used to represent the final AOD for each sample.

**Table 1.** Clinical characteristics of 20 T2DM patients with or without MASLD for DirectDIA mass spectrometry.

Variables	T2DM (*n* = 10)	T2DM + MASLD (*n* = 10)	Effect Size (95% CI)	*P* value
Gender			1.000 (0.173, 5.772)	1.000
Male (%)	5 (50%)	5 (50%)		
Female (%)	5 (50%)	5 (50%)		
Age (y)	46.40 ± 17.70	45.80 ± 14.38	−0.600 (−15.753, 14.553)	0.935
Diabetes duration (m)	0.50 (0.25, 2.00)	0.38 (0.25, 2.25)	0.000 (−0.750, 1.000)	1.000
Hypertension (%)	2 (20%)	1 (10%)	2.250 (0.170, 29.767)	1.000
BMI (kg/m^2^)	23.87 ± 1.84	25.10 ± 2.13	1.234 (−0.637, 3.105)	0.183
FPG (mmol/L)	11.08 ± 3.78	11.31 ± 2.61	0.233 (−2.819, 3.285)	0.874
HbA1c (%)	10.52 ± 2.24	9.64 ± 1.89	−0.880 (−2.828, 1.068)	0.880
ALB (g/L)	42.08 ± 2.83	44.04 ± 2.91	1.960 (−0.736, 4.656)	0.144
ALT (U/L)	23.00 (10.75, 31.25)	23.00 (14.63, 30.75)	1.000 (−10.000, 14.000)	0.791
AST (U/L)	20.50 (12.00, 24.25)	20.13 (17.08, 37.25)	5.000 (−3.900, 13.000)	0.307
ALP (U/L)	80.00 ± 22.17	80.39 ± 17.73	0.390 (−17.394, 21.816)	0.815
GGT (U/L)	16.50 (12.50, 20.50)	25.50 (21.16, 74.50)	11.500 (3.000, 38.000)	0.024
TG (mmol/L)	1.06 (0.83, 1.24)	1.76 (1.01, 3.20)	0.605 (0.100, 1.590)	0.046
TCH (mmol/L)	4.72 (4.32, 5.20)	5.03 (3.87, 6.07)	0.315 (−0.780, 1.240)	0.762
LDL-C (mmol/L)	3.09 ± 0.98	2.98 ± 0.98	−0.111 (−1.028, 0.806)	0.802
HDL-C (mmol/L)	1.10 ± 0.35	1.03 ± 0.33	−0.065 (−0.385, 0.255)	0.675
Cre (μmol/L)	52.22 ± 12.64	50.00 ± 12.40	−2.220 (−12.890, 10.278)	0.815
Hb (g/L)	141.78 ± 12.06	144.10 ± 13.76	2.322 (−10.270, 14.914)	0.702

Note: Continuous variables conforming to a normal distribution are presented as mean ± SD and compared using the independent samples t-test, with the mean difference and its 95% CI reported. Variables not conforming to a normal distribution are presented as median (IQR) and compared using the Mann-Whitney U test, with the Hodges–Lehmann estimator of the median difference and its 95% CI reported. Categorical variables are presented as number (percentage) and compared using the chi-square test, with the odds ratio and its 95% CI reported.

T2DM: Type 2 Diabetes Mellitus; MASLD: Metabolic Dysfunction-associated Steatotic Liver Disease; DIA: Data-independent Acquisition; CI: confidence interval; BMI: Body Mass Index; FPG: Fasting Plasma Glucose; HbA1c: Glycated Haemoglobin; ALB: Albumin; ALT: Alanine Aminotransferase; AST: Aspartate Aminotransferase; ALP: Alkaline Phosphatase; GGT; Gamma-Glutamyl Transpeptidase; TG; Triglycerides; TCH; Total Cholesterol; LDL-C; Low-Density Lipoprotein Cholesterol; HDL-C; High-Density Lipoprotein Cholesterol; Cre; Creatinine; Hb; Hemoglobin.

### Statistical analysis

Data analysis was performed using SPSS software version 25.0, and graphs were generated using GraphPad Prism 9.0. Quantitative data that followed a normal distribution were presented as mean ± standard deviation (SD) and compared between groups using a Student’s independent t-test. Non-normally distributed quantitative data were presented as medians [interquartile range (IQR): P25, P75], and differences between groups were assessed using the Mann-Whitney U test. Categorical data were presented as frequencies or percentages (%) and compared using the chi-square test. Spearman correlation analysis was employed to evaluate the relationship between XPNPEP3 expression levels and clinical data. Univariate logistic regression analysis was used to identify factors associated with MASLD in T2DM patients. To assess the independent association between plasma XPNPEP3 and MASLD, a multivariate logistic regression model was constructed. Due to the limited sample size and the need to avoid overfitting, covariates (age, gender, BMI, and diabetes duration) were selected based on their known clinical and biological relevance rather than on the results of univariate analyses. Additionally, receiver operating characteristic (ROC) curve analysis was performed to assess the diagnostic sensitivity and specificity of plasma XPNPEP3 for MASLD, with the area under the curve (AUC) calculated. A *P*-value of less than 0.05 was considered statistically significant.

## Results

### General information on the twenty subjects subjected to directDIA mass spectrometry

The clinical and biochemical characteristics of the 20 patients who underwent plasma proteomic testing are summarized in Table 1. The levels of TG and GGT in T2DM patients with MASLD were significantly higher than those in T2DM patients without MASLD (*p* < 0.05), while other indicators such as gender, age, diabetes duration, BMI, and FPG showed no significant differences between the two groups. Consequently, the two sequenced populations were well-matched in terms of demographic and most metabolic parameters.

### Identification of total proteins in plasma and screening for DEPs

**Figure 2.**
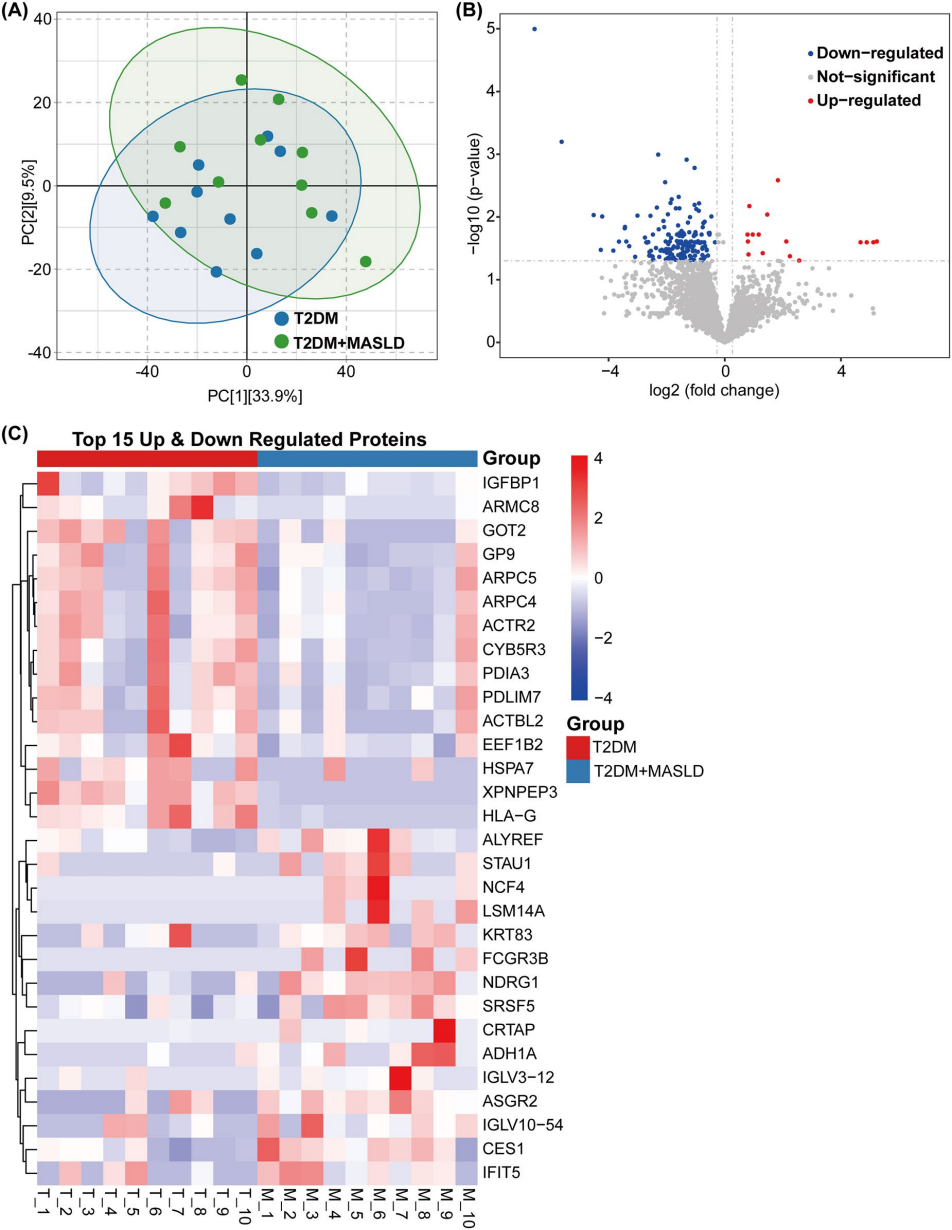
DEPs were identified through proteomic sequencing of peripheral blood plasma from 10 T2DM patients with MASLD and 10 without, using |log_2_FoldChange| ≥ 0.25 and *p* < 0.05 as screening criteria. (A) PCA. (B) Volcano plot displaying DEPs between T2DM patients with and without MASLD. (C) Cluster diagram illustrating the top 15 upregulated and top 15 downregulated DEPs in T2DM patients with and without MASLD. T2DM: Type 2 Diabetes Mellitus; MASLD: Metabolic Dysfunction-associated Steatotic Liver Disease; PCA: Principal Component Analysis; DEPs: Differentially Expressed Proteins.

In plasma samples from the 20 patients, a total of 3152 proteins were identified (unique peptides ≥ 2, protein FDR ≤ 0.01). PCA showed some overlap between the T2DM and T2DM + MASLD groups, although a distinct separation trend was evident (Figure 2A), indicating potential metabolic differences. These proteins were selected for DEPs screening, with the criteria set as *P*-value < 0.05 and fold change ≤ 0.83 or ≥ 1.2, based on Student’s t-test or chi-square test [40]. Quantitative analysis revealed 176 DEPs in the plasma of T2DM patients with MASLD compared to those without MASLD, with statistically significant differences (*p* < 0.05). Of these, 16 proteins were upregulated, and 160 were downregulated (Table SII; Figure 2B), with XPNPEP3 showing the most significant fold change among the downregulated proteins. The heatmap of the top 15 upregulated and top 15 downregulated proteins revealed distinct plasma protein expression patterns between the two groups (Figure 2C). The complete heatmap of all DEPs is available in the (Supplementary Materials Figure S1).

**Table 2.** Comparisons of clinical and biochemical characteristics between the two groups.

Variables	T2DM (*n* = 42)	T2DM + MASLD (*n* = 42)	Effect Size (95% CI)	*P* value
Gender			0.909 (0.385, 2.143)	0.827
Male	22 (52.4%)	23 (54.8%)		
Female	20 (47.6%)	19 (45.2%)		
Age (y)	51.00 (42.00, 58.25)	47.50 (36.75, 55.25)	−5.000 (−12.000, −2.000)	0.010
Diabetes duration (m)	42.00 (8.50, 84.00)	96.00 (12.00, 123.00)	36.000 (11.500, 78.000)	0.001
Hypertension	18 (42.9%)	23 (54.8%)	0.620 (0.262, 1.467)	0.275
T2DM therapy			1.734 (0.686, 4.382)	0.236
Diet therapy	16 (38.1%)	11 (26.2%)		
Diet therapy + medications*	26 (61.9%)	31 (73.8%)		
BMI (kg/m^2^)	24.81 ± 2.18	25.72 ± 1.76	0.913 (0.054, 1.772)	0.038
FPG (mmol/L)	8.02 (6.72, 11.54)	8.24 (7.10, 12.16)	0.220 (−0.900, 1.650)	0.768
HbA1c (%)	9.17 ± 2.27	9.30 ± 2.10	0.127 (−0.823, 1.077)	0.791
ALB (g/L)	41.80 ± 3.27	43.58 ± 3.99	1.779 (0.194, 3.363)	0.028
ALT (U/L)	15.50 (12.00, 23.25)	25.45 (17.15, 42.08)	9.000 (4.000, 14.000)	<0.001
AST (U/L)	17.00 (13.75, 20.25)	20.75 (17.00, 28.00)	4.000 (2.000, 7.000)	0.001
ALP (U/L)	81.00 (64.75, 93.00)	90.50 (70.50, 111.40)	12.000 (0.020, 23.000)	0.040
GGT (U/L)	19.50 (13.75, 29.00)	28.50 (16.00, 49.00)	7.000 (1.000, 15.000)	0.020
TG (mmol/L)	1.14 (0.92, 1.63)	1.78 (1.43, 2.42)	0.630 (0.360, 0.910)	<0.001
TCH (mmol/L)	4.32 (3.78, 5.12)	4.73 (4.34, 5.60)	0.493 (0.060, 0.930)	0.021
LDL-C (mmol/L)	3.04 (2.35, 3.66)	3.14 (2.68, 4.14)	0.260 (−0.160, 0.663)	0.212
HDL-C (mmol/L)	1.12 (0.85, 1.30)	1.12 (0.88, 1.24)	−0.010 (−0.150, 0.110)	0.907
Cre (μmol/L)	61.09 ± 16.78	59.37 ± 11.88	−1.721 (−8.041, 4.600)	0.525
Hb (g/L)	139.38 ± 15.40	146.72 ± 14.17	7.338 (0.915, 13.761)	0.026
XPNPEP3 (pg/mL)	47.75 (45.70, 49.01)	44.72 (42.80, 45.90)	−3.169 (−4.200, −1.922)	<0.001

Note: Continuous variables conforming to a normal distribution are presented as mean ± SD and compared using the independent samples t-test, with the mean difference and its 95% CI reported. Variables not conforming to a normal distribution are presented as median (IQR) and compared using the Mann-Whitney U test, with the Hodges–Lehmann estimator of the median difference and its 95% CI reported. Categorical variables are presented as number (percentage) and compared using the chi-square test, with the odds ratio and its 95% CI reported. *Medications indicate antidiabetic drugs other than those listed in the exclusion criteria (e.g. sulfonylureas, α-glucosidase inhibitors, insulin).

T2DM: Type 2 Diabetes Mellitus; MASLD: Metabolic Dysfunction-associated Steatotic Liver Disease; CI: confidence interval; BMI: Body Mass Index; FPG: Fasting Plasma Glucose; HbA1c: Glycated Haemoglobin; ALB: Albumin; ALT: Alanine Aminotransferase; AST: Aspartate Aminotransferase; ALP: Alkaline Phosphatase; GGT: Gamma-Glutamyl Transpeptidase; TG: Triglycerides; TCH: Total Cholesterol; LDL-C: Low-Density Lipoprotein Cholesterol; HDL-C: High-Density Lipoprotein Cholesterol; Cre: Creatinine; Hb: Hemoglobin.

### Functional enrichment and bioinformatic analysis of DEPs

**Figure 3.**
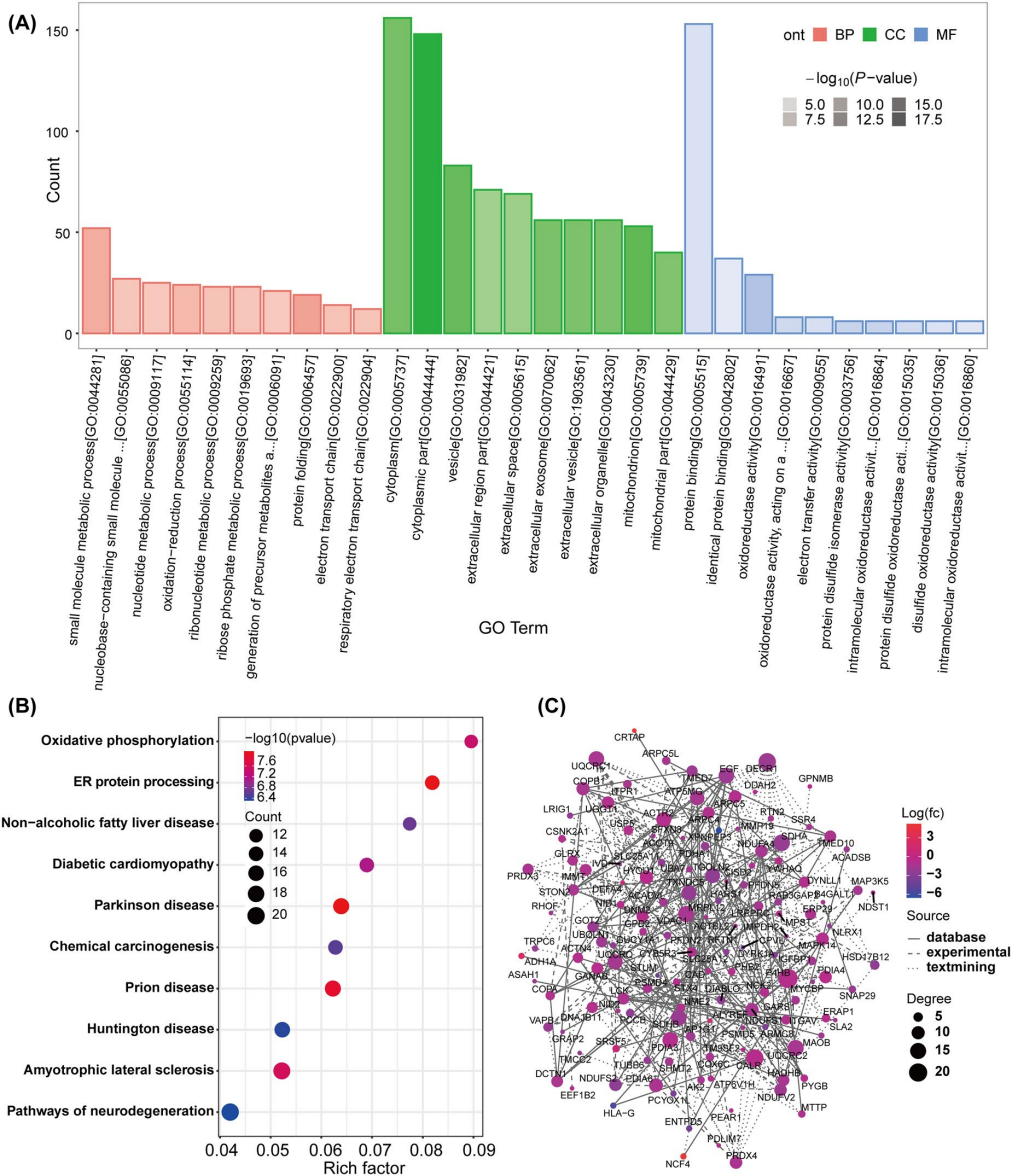
Functional enrichment and bioinformatic analysis of DEPs. (A) GO enrichment analysis of DEPs. (B) KEGG enrichment analysis of DEPs. The rich factor is calculated and displayed on the horizontal axis. The size of the dots represents the number of proteins, and the color of the dots indicates the *P*-values. (C) PPInetwork analysis of DEPs between T2DM patients with and without MASLD. Distinct dot colors represent the expression levels of DEPs, the size of the dots indicates the connectivity degree of DEPs, and the type of connection reflects the source of the interaction relationship. DEPs: Differentially Expressed Proteins; GO: Gene Ontology; KEGG: Kyoto Encyclopedia of Genes and Genomes; PPI: Protein-Protein Interaction; BP: Biological Process; CC: Cellular Component; MF: Molecular Function.

GO analysis of the identified DEPs (Figure 3A) highlighted the major biological functions (BP), cellular components (CC), and molecular functions (MF) associated with these proteins. In terms of BP, the most significantly enriched categories were related to metabolic activities, particularly small molecule metabolic processes, nucleotide metabolic processes, and redox processes. Protein folding was also identified as a key function. In the CC category, DEPs were predominantly localized in extracellular vesicles/exosomes and mitochondria, with additional enrichment in the cytoplasmic fraction. Regarding MF, the enriched entries were consistent with the observed biological processes, particularly oxidoreductase activity and electron transfer functions. A set of entries related to protein disulfide isomerase and oxidoreductase activities was also significantly enriched. Together, these findings suggest that the co-occurrence of MASLD and T2DM impacts cellular metabolic processes, redox homeostasis, and intercellular communication through extracellular vesicles.

To further investigate the systemic pathways affected by MASLD in the context of T2DM, KEGG pathway enrichment analysis was conducted. As shown in Figure 3B, the most significantly enriched pathway was oxidative phosphorylation (hsa00190), the primary process for ATP production in mitochondria. The second most enriched pathway was protein processing in the endoplasmic reticulum (ER) (hsa04141), suggesting potential involvement of ER stress. Additionally, several disease-specific pathways were significantly enriched. Nonalcoholic fatty liver disease (hsa04932) was notably enriched, aligning with the phenotype of our subjects and providing molecular validation for the model. The enrichment of diabetic cardiomyopathy (hsa05415) indicates a potential connection between hepatic steatosis and cardiac complications in diabetes. Interestingly, neurodegenerative disease pathways, including Parkinson’s disease, Huntington’s disease, and amyotrophic lateral sclerosis, were also significantly enriched, implying that the metabolic disorders in T2DM and MASLD may share common pathological features with neurodegenerative diseases.

PPI network analysis (Figure 3C, Table SIII) was used to identify core molecules and functional modules within the DEPs. The network highlighted P4HB as the central hub with the highest degree of connectivity. A significant module containing several subunits of the mitochondrial respiratory chain complex (e.g. UQCRC1, UQCRC2, NDUFS1, NDUFS2, SDHA, and SDHB) was identified as a mitochondrial energy metabolism module primarily involved in oxidative phosphorylation and the electron transport chain. This supports the findings from our KEGG analysis, reinforcing the central role of oxidative phosphorylation. These results suggest that mitochondrial energy dysfunction and heightened oxidative stress are central mechanisms in the development and progression of MASLD in T2DM patients. Furthermore, another important cluster involved in protein folding, disulfide bond formation, and ER stress management included P4HB, CALR, PDIA3, PDIA4, PDIA6, and GANAB. Other notable hubs in the network included ITPR1 (a key calcium release channel on the ER membrane), PRDX3 and PRDX4 (mitochondrial and ER-associated antioxidant enzymes), and ACTR2 (involved in cytoskeletal organization and vesicular trafficking). The widespread downregulation of these core proteins suggests that key organelle functions, particularly those of the mitochondria and ER, are impaired in T2DM patients with MASLD.

**Table 3.** Correlation analysis of XPNPEP3 with clinical and biochemical parameters.

Variables	All Patients (*n* = 84)		T2DM (*n* = 42)		T2DM + MASLD (*n* = 42)	
	r (95% CI)	*P* value	r (95% CI)	*P* value	r (95% CI)	*P* value
Gender	0.080 (−0.130, 0.294)	0.469	0.047 (-0.256, 0.370)	0.769	0.188 (-0.110, 0.501)	0.233
Age (y)	0.295 (0.105, 0.467)	0.006	0.111 (-0.175, 0.400)	0.485	−0.026 (-0.308, 0.285)	0.869
Diabetes duration (m)	−0.334 (-0.559, −0.189)	0.002	−0.223 (-0.439, 0.146)	0.156	−0.198 (-0.387, 0.259)	0.209
BMI (kg/m^2^)	−0.127 (−0.340, 0.041)	0.251	0.197 (-0.146, 0.515)	0.211	0.058 (-0.241, 0.379)	0.714
FPG (mmol/L)	−0.039 (−0.246, 0.158)	0.723	−0.175 (-0.468, 0.138)	0.268	0.141 (-0.151, 0.449)	0.374
HbA1c (%)	−0.095 (−0.305, 0.141)	0.390	−0.181 (-0.504, 0.146)	0.252	−0.085 (-0.325, 0.253)	0.594
ALB (g/L)	−0.224 (−0.436, −0.009)	0.041	−0.007 (-0.336, 0.318)	0.966	−0.064 (-0.389, 0.242)	0.688
ALT (U/L)	−0.331 (−0.502, −0.137)	0.002	0.014 (-0.190, 0.392)	0.932	0.003 (-0.315, 0.331)	0.987
AST (U/L)	−0.244 (−0.430, −0.048)	0.025	0.168 (-0.134, 0.483)	0.287	0.126 (-0.191, 0.436)	0.426
ALP (U/L)	−0.231 (−0.425, −0.024)	0.035	−0.176 (-0.448, 0.128)	0.264	0.128 (-0.314, 0.367)	0.420
GGT (U/L)	−0.254 (−0.462, −0.044)	0.020	−0.092 (-0.400, 0.186)	0.561	−0.084 (-0.395, 0.222)	0.598
TG (mmol/L)	−0.409 (−0.601, −0.200)	<0.001	−0.042 (-0.373, 0.254)	0.790	0.023 (-0.309, 0.358)	0.887
TCH (mmol/L)	−0.183 (−0.384, 0.025)	0.095	0.107 (-0.209, 0.386)	0.499	0.021 (-0.275, 0.302)	0.893
LDL-C (mmol/L)	−0.116 (−0.344, 0.092)	0.295	0.025 (-0.287, 0.347)	0.876	−0.019 (-0.310, 0.280)	0.907
HDL-C (mmol/L)	0.095 (−0.131, 0.299)	0.391	0.220 (-0.077, 0.501)	0.161	0.097 (-0.285, 0.400)	0.542
Cre (μmol/L)	−0.015 (−0.208, 0.216)	0.890	0.040 (-0.296, 0.364)	0.799	−0.383 (-0.624, −0.016)	0.012
Hb (g/L)	−0.165 (−0.366, 0.026)	0.134	0.114 (-0.234, 0.412)	0.471	0.058 (-0.223, 0.364)	0.717

**Notes:** Data are presented as Spearman’s rank correlation coefficient (*r*) with 95% CI calculated using the percentile bootstrap method (1000 resamples). *P* values are two-tailed.

T2DM: Type 2 Diabetes Mellitus; MASLD: Metabolic Dysfunction-associated Steatotic Liver Disease; CI: confidence interval; BMI: Body Mass Index; FPG: Fasting Plasma Glucose; HbA1c: Glycated Haemoglobin; ALB: Albumin; ALT: Alanine Aminotransferase; AST: Aspartate Aminotransferase; ALP; Alkaline Phosphatase; GGT; Gamma-Glutamyl Transpeptidase; TG; Triglycerides; TCH; Total Cholesterol; LDL-C: Low-Density Lipoprotein Cholesterol; HDL-C: High-Density Lipoprotein Cholesterol; Cre: Creatinine; Hb: Hemoglobin.

### Baseline characteristics comparison among the two groups

**Figure 4.**
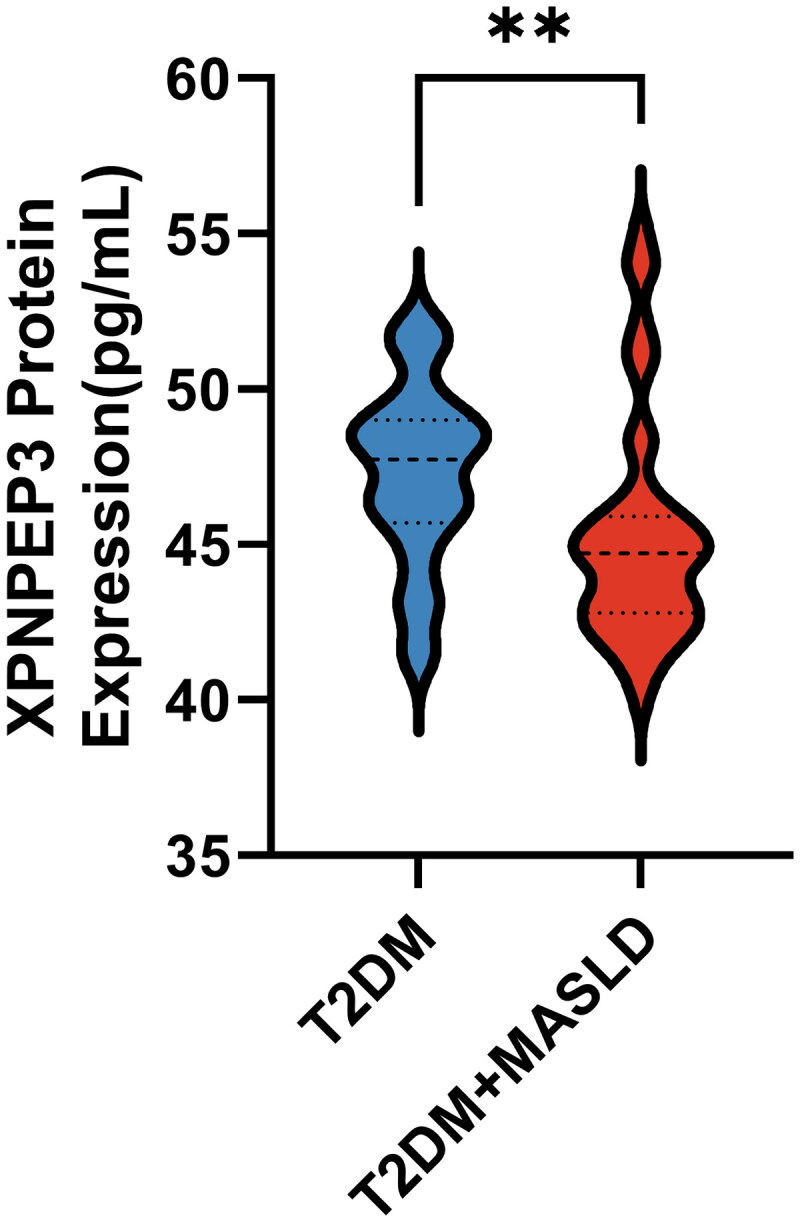
Plasma XPNPEP3 protein expression levels of two study groups (*n* = 42). Differences between the two groups were analyzed using Mann-Whitney U-test. Significance levels: **p < 0.001.

A total of 56 T2DM patients without MASLD and 59 T2DM patients with MASLD were initially screened, with 84 participants included in the final analysis. These were divided into two groups: the T2DM group (*n* = 42) and the T2DM + MASLD group (*n* = 42) (Figure 1). Table 2 summarizes the clinical and biochemical characteristics of the two groups. No significant differences were observed between the groups in sex distribution (*p* = 0.827), hypertension prevalence (*p* = 0.275), or the distribution of T2DM therapy (*p* = 0.236), indicating good matching between the groups. Patients in the T2DM + MASLD group were younger (*p* = 0.010), had a longer duration of diabetes (*p* = 0.001), and a higher BMI (*p* = 0.038) compared to those in the T2DM group. No significant differences were observed in FPG, HbA1c, LDL-C, HDL-C, or Cre levels between the two groups (*p* > 0.05). Notably, ALB (*p* = 0.028), ALT (*p* < 0.001), AST (*p* = 0.001), ALP (*p* = 0.040), GGT (*p* = 0.020), TG (*p* < 0.001), TCH (*p* = 0.021), and Hb (*p* = 0.026) levels were significantly higher in the T2DM + MASLD group compared to the T2DM group. Conversely, plasma XPNPEP3 levels were significantly lower in the T2DM + MASLD group (*p* < 0.001), a difference that is clearly depicted in Figure 4.

### Correlation of XPNPEP3 levels with other parameters

Table 3 presents the results of the correlation analysis between plasma XPNPEP3 levels and various clinical and biochemical indicators. In the overall study population (*n* = 84), plasma XPNPEP3 levels were positively correlated with age (*r* = 0.295, *p* = 0.006). XPNPEP3 was also negatively correlated with the duration of diabetes (r = −0.334, *p* = 0.002), ALB (r = −0.224, *p* = 0.041), ALT (r = −0.331, *p* = 0.002), AST (r = −0.244, *p* = 0.025), ALP (r = −0.231, *p* = 0.035), GGT (r = −0.254, *p* = 0.020), and TG (r = −0.409, *p* < 0.001). No significant correlation was observed between XPNPEP3 and other metabolic indicators, such as BMI, FPG, HbA1c, TCH, LDL-C, HDL-C, and Cre (*p* > 0.05). Additionally, in the T2DM + MASLD group, XPNPEP3 levels were negatively correlated with Cre levels (r = −0.383, *p* = 0.012).

### Univariate logistic regression analysis of related factors

Univariate logistic regression analysis identified several factors significantly associated with the presence of MASLD in T2DM patients (Table 4). Advanced age was inversely associated with MASLD (OR = 0.941, 95% CI: 0.902–0.982, *p* = 0.006). Conversely, longer duration of diabetes was associated with a higher likelihood of MASLD (OR = 1.015, 95% CI: 1.006–1.024, *p* = 0.001). Higher BMI (OR = 1.269, 95% CI: 1.009–1.596, *p* = 0.042), ALB (OR = 1.147, 95% CI: 1.011–1.302, *p* = 0.033), ALT (OR = 1.096, 95% CI: 1.039–1.156, *p* = 0.001), AST (OR = 1.170, 95% CI: 1.062–1.289, *p* = 0.001), TG (OR = 1.231, 95% CI: 1.026–1.817, *p* = 0.001), TCH (OR = 1.814, 95% CI: 1.126–2.122, *p* = 0.014), and Hb (OR = 1.034, 95% CI: 1.003–1.067, *p* = 0.029) were all positively associated with MASLD. Notably, higher plasma XPNPEP3 levels were significantly associated with a lower likelihood of MASLD (OR = 0.757, 95% CI: 0.644–0.890, *p* = 0.001). Other variables, such as gender, FPG, HbA1c, ALP, GGT, LDL-C, HDL-C, and Cre, were not significantly associated with MASLD (*p* > 0.05).

**Table 4.** Univariate logistic regression analysis of factors associated with MASLD in T2DM.

Variables	β	OR with 95% CI	*P* value
Gender	0.096	1.100 (0.467, 2.595)	0.827
Age (y)	−0.060	0.941 (0.902, 0.982)	0.006
Diabetes duration (m)	0.015	1.015 (1.006, 1.024)	0.001
BMI (kg/m^2^)	0.238	1.269 (1.009, 1.596)	0.042
FPG (mmol/L)	0.016	1.016 (0.893, 1.156)	0.808
HbA1c (%)	0.027	1.028 (0.843, 1.252)	0.788
ALB (g/L)	0.137	1.147 (1.011, 1.302)	0.033
ALT (U/L)	0.092	1.096 (1.039, 1.156)	0.001
AST (U/L)	0.157	1.170 (1.062, 1.289)	0.001
ALP (U/L)	0.012	1.012 (0.996, 1.028)	0.134
GGT (U/L)	0.020	1.020 (0.999, 1.042)	0.067
TG (mmol/L)	1.443	1.231 (1.026, 1.817)	0.001
TCH (mmol/L)	0.595	1.814 (1.126, 2.122)	0.014
LDL-C (mmol/L)	0.406	1.501 (0.927, 2.131)	0.099
HDL-C (mmol/L)	−0.501	0.606 (0.181, 1.233)	0.417
Cre (μmol/L)	−0.008	0.992 (0.963, 1.022)	0.584
Hb (g/L)	0.034	1.034 (1.003, 1.067)	0.029
XPNPEP3 (pg/mL)	−0.279	0.757 (0.644, 0.890)	0.001

Notes: Univariate logistic regression analysis was performed in the validation cohort (*n* = 84), which included 42 patients with T2DM alone and 42 patients with T2DM and MASLD.

OR: Odds Ratio; CI: Confidence Interval; T2DM: Type 2 Diabetes Mellitus; MASLD: Metabolic Dysfunction-associated Steatotic Liver Disease; BMI: Body Mass Index; FPG: Fasting Plasma Glucose; HbA1c: Glycated Haemoglobin; ALB: Albumin; ALT: Alanine Aminotransferase; AST: Aspartate Aminotransferase; ALP: Alkaline Phosphatase; GGT: Gamma-Glutamyl Transpeptidase; TG: Triglycerides; TCH: Total Cholesterol; LDL-C: Low-Density Lipoprotein Cholesterol; HDL-C: High-Density Lipoprotein Cholesterol; Cre: Creatinine; Hb: Hemoglobin.

### Multivariable analysis of factors independently associated with MASLD in T2DM

The results of the multivariable logistic regression analysis are presented in Table 5. After adjustment for gender, age, diabetes duration, and BMI, lower plasma levels of XPNPEP3 remained independently and inversely associated with the presence of MASLD (OR = 0.825, 95% CI: 0.695–0.980, *p* = 0.028). Among the adjusted covariates, longer diabetes duration (OR = 1.017, 95% CI: 1.005–1.029, *p* = 0.007) and higher BMI (OR = 1.215, 95% CI: 1.038–1.574, *p* = 0.039) were also significantly associated with an increased likelihood of MASLD. In this model, neither gender nor age showed a statistically significant association with MASLD.

**Table 5.** Multivariable logistic regression analysis of factors associated with MASLD in T2DM.

Variables	β	OR with 95% CI	*P* value
Gender	−0.586	0.557 (0.173,1.789)	0.325
Age (y)	−0.074	0.928 (0.875,1.085)	0.054
Diabetes duration (m)	0.016	1.017 (1.005, 1.029)	0.007
BMI (kg/m^2^)	0.195	1.215 (1.038, 1.574)	0.039
XPNPEP3 (pg/mL)	−0.192	0.825 (0.695, 0.980)	0.028

Notes: Multivariable logistic regression analysis was performed in the validation cohort (*n* = 84), which included 42 patients with T2DM alone and 42 patients with T2DM and MASLD.

OR: Odds Ratio; CI: Confidence Interval; T2DM: Type 2 Diabetes Mellitus; MASLD: Metabolic Dysfunction-associated Steatotic Liver Disease; BMI: Body Mass Index.

### Diagnostic performance of XPNPEP3 in peripheral blood for MASLD in T2DM

**Figure 5.**
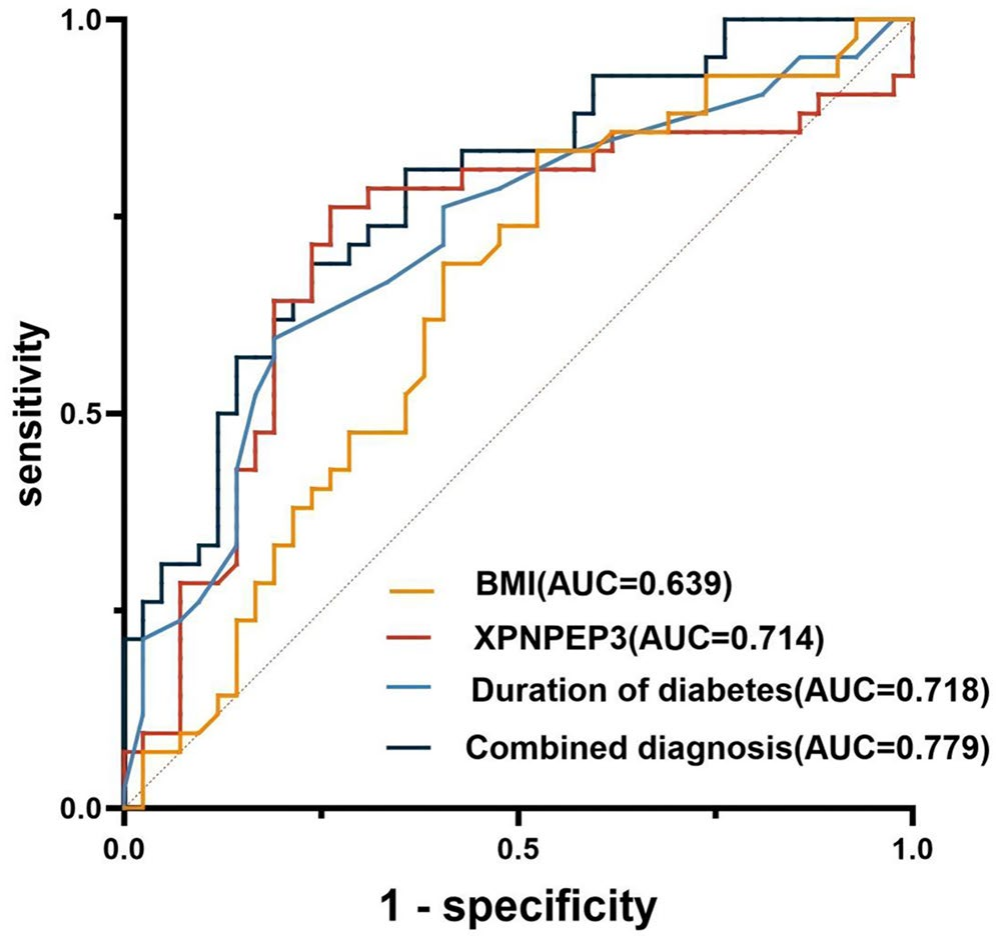
The diagnostic value of plasma XPNPEP3 levels, in combination with other clinical indicators, for distinguishing between the T2DM group and the T2DM + MASLD group was assessed using ROC curve analysis. Plasma XPNPEP3 demonstrated a sensitivity of 80.1%, specificity of 73.8%, and an AUC of 0.714 (95% CI: 0.597–0.830, *p* = 0.001). For BMI and diabetes duration, sensitivity values were 83.3% and 59.5%, specificity values were 47.6% and 80.9%, and AUCs were 0.639 (95% CI: 0.519–0.758, *p* = 0.028) and 0.718 (95% CI: 0.607–0.829, *p* = 0.001), respectively. The combined AUC for all three factors was 0.779 (95% CI: 0.681–0.877, *p* = 0.001).

The ROC curve was used to assess the diagnostic efficacy of each indicator for T2DM patients with MASLD. As shown in Figure 5 and Table 6, the AUC for plasma XPNPEP3 levels was 0.714 (95% CI: 0.597–0.830). At the optimal cut-off value of 45.98 pg/mL, the sensitivity was 80.1%, and the specificity was 73.8%. The AUCs for diabetes duration and BMI as factors associated with MASLD in T2DM patients were 0.718 (95% CI: 0.607–0.829) and 0.639 (95% CI: 0.519–0.758), respectively, with sensitivities of 59.5% and 83.3%, and specificities of 80.9% and 47.6%. A combined predictive model incorporating XPNPEP3, BMI, and diabetes duration had an AUC of 0.779 (95% CI: 0.681–0.877), with a sensitivity of 80.9% and a specificity of 64.2%. This analysis indicated that the combination of XPNPEP3 with various indicators, such as diabetes duration and BMI, provided superior diagnostic value for T2DM patients with MASLD compared to individual factors (*p* < 0.05).

**Table 6.** Diagnostic performance of individual and combined indicators for MASLD in T2DM.

Index	AUC	95%CI	Optimal critical value	Sensitivity	Specificity	*P* value
BMI (kg/m^2^)	0.639	0.519- 0.758	24.35	0.833	0.476	0.028
XPNPEP3 (pg/mL)	0.714	0.597- 0.830	45.98	0.801	0.738	0.001
Duration of diabetes (m)	0.718	0.607- 0.829	10.50	0.595	0.809	0.001
Combined diagnosis	0.779	0.681- 0.877	–	0.809	0.642	0.001

Notes: The combined model included BMI, XPNPEP3, and duration of diabetes.

T2DM: Type 2 Diabetes Mellitus; MASLD: Metabolic Dysfunction-associated Steatotic Liver Disease; BMI: Body Mass Index; AUC: Area Under The Curve; CI: Confidence Interval.

### Immunohistochemical analysis of XPNPEP3 protein in liver tissue

**Figure 6.**
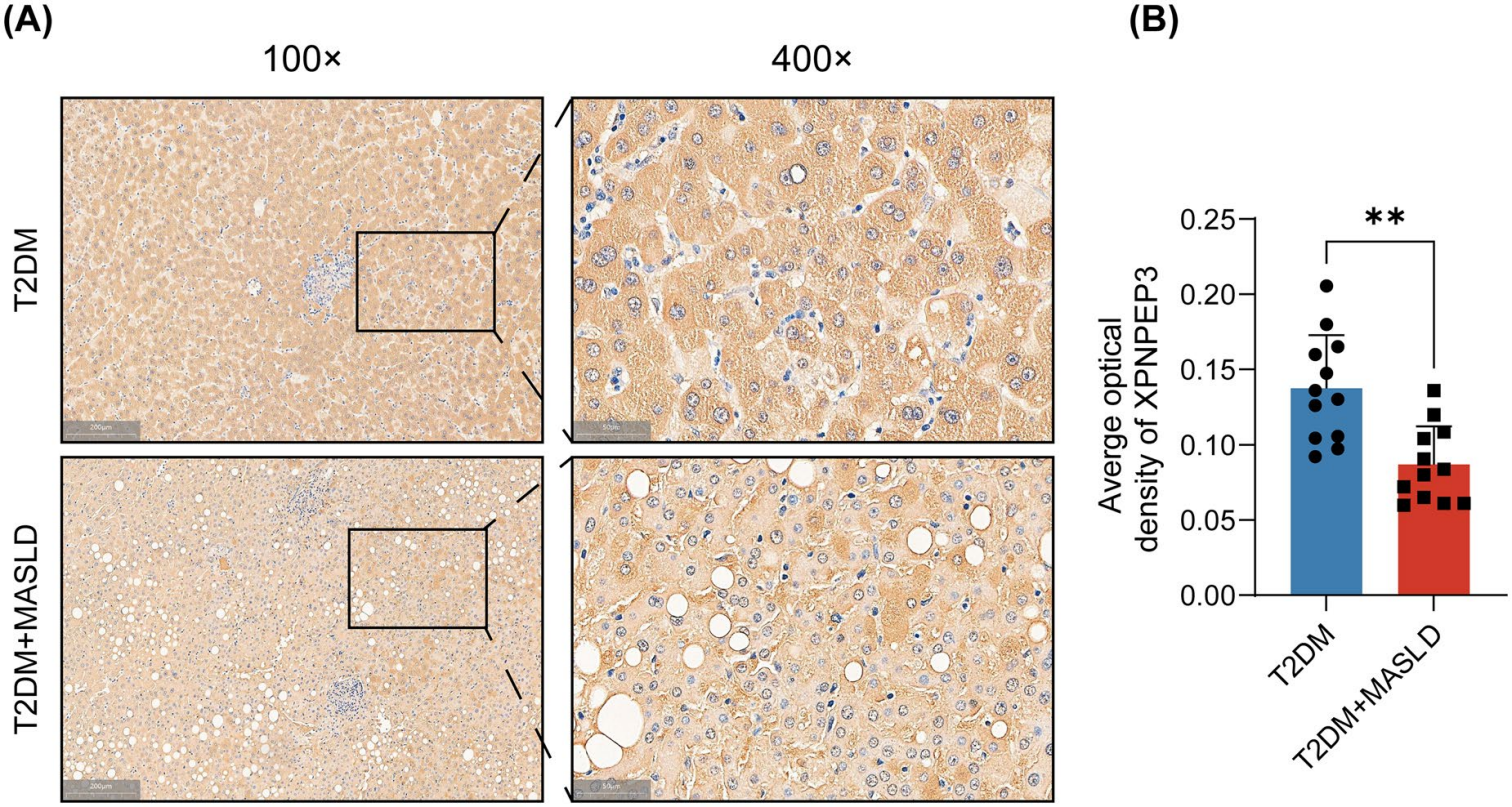
Immunohistochemical analysis of XPNPEP3 expression in liver tissues. (A) Representative images of XPNPEP3 immunohistochemical staining in the T2DM group (upper panels) and T2DM + MASLD group (lower panels). Brown indicates XPNPEP3-positive staining, and blue indicates nuclear counterstain with hematoxylin. Magnification: left panels, ×100; right panels, ×400. (B) Quantitative analysis of XPNPEP3 staining intensity expressed as average optical density. Differences between the two groups (*n* = 12) were analyzed using Mann-Whitney U-test. Significance levels: **p < 0.001.

To validate these findings at the target organ level, XPNPEP3 protein expression in liver tissue was examined through immunohistochemistry. As shown in Figure 6A, XPNPEP3 staining was visibly weaker in liver tissues from T2DM patients with MASLD compared to those without MASLD. Quantitative analysis of staining intensity, expressed as AOD, revealed that XPNPEP3 expression was significantly lower in the T2DM + MASLD group (*n* = 12) than in the T2DM group (*n* = 12) (Figure 6B, *p* < 0.001). These results confirmed that XPNPEP3 protein expression was downregulated in the liver of T2DM patients with MASLD, consistent with the observed decrease in plasma levels, and strongly support the role of XPNPEP3 in the pathogenesis of T2DM with MASLD.

## Discussion

T2DM and MASLD have attracted increasing attention globally due to their significant impact on public health and quality of life. Both conditions are becoming more prevalent worldwide, with a potential link through IR. The coexistence of T2DM and MASLD exacerbates the risk of T2DM-related complications and accelerates the progression of MASLD to MASH, cirrhosis, and even liver cancer [43]. Given the high prevalence of T2DM and the increasing incidence of MASLD in this patient population, along with their shared pathophysiological features, it is critical to explore the molecular characteristics of MASLD in T2DM patients to identify potential biomarkers and develop novel diagnostic and therapeutic strategies.

In this study, directDIA proteomics was employed to analyze the plasma proteomic profiles of T2DM patients with and without MASLD. A total of 176 DEPs were identified, with XPNPEP3 being the most significantly downregulated protein. Furthermore, the expression of XPNPEP3 was significantly lower in T2DM patients with MASLD compared to those without MASLD. XPNPEP3 levels were also found to be negatively correlated with key metabolic parameters, including liver injury markers and TG levels. Multivariable logistic regression analysis revealed that lower plasma XPNPEP3 levels were independently associated with the presence of MASLD in T2DM patients. ROC curve analysis indicated its value in distinguishing MASLD, particularly when combined with clinical parameters such as BMI and diabetes duration, which improved the discriminatory ability. Additionally, downregulation of XPNPEP3 expression in liver tissues of T2DM patients with MASLD was validated at the target organ level. Therefore, reduced XPNPEP3 expression is significantly associated with MASLD in T2DM and shows promise as a potential biomarker to aid in the identification of this condition. To the best of our knowledge, this is the first study to investigate the relationship between XPNPEP3 expression changes and the pathogenesis of MASLD in T2DM patients.

In the previous study, a total of 3152 proteins were identified in plasma samples using proteomics, demonstrating the high dynamic range and reproducibility of protein quantification using directDIA technology [19]. PCA revealed a separation trend between the T2DM and T2DM + MASLD groups, although some overlap remained, indicating systemic differences in peripheral blood plasma protein expression between the two groups. The variation observed within each group highlights the heterogeneity of the MASLD patient population, which is influenced by primary drivers and coexisting disease regulators [44]. Thus, in clinical practice, relying solely on proteomic data may not be sufficient to fully distinguish between disease states, and integrating other clinical indicators is essential to enhance diagnostic accuracy. Among the DEPs, the majority were downregulated in T2DM patients with MASLD, suggesting that protein expression and metabolic pathways are broadly suppressed in the context of the dual metabolic disorders resulting from the coexistence of these two diseases.

In the subsequent GO enrichment analysis, the biological characteristics of DEPs were examined from three perspectives: biological processes, cellular components, and molecular functions. At the BP level, significant enrichment was observed in small molecule metabolic processes, nucleotide metabolic processes, and redox processes, reflecting the systemic metabolic dysfunction present in the MASLD state [45]. Notably, the redox process dysfunction is particularly striking, as oxidative stress is a critical mechanism driving the progression of MASLD [46]. Additionally, the enrichment of DEPs in the protein folding process suggests activation of ER stress, indicating significant disturbance in cellular ER function. The liver, being the primary organ for synthesizing plasma proteins [47], experiences general inhibition of protein translation, which likely reflects a severe stress state in hepatocytes, contributing to the accelerated progression of liver injury in T2DM patients with MASLD. At the CC level, DEPs were significantly enriched in extracellular vesicles/exosomes and mitochondrial components. The enrichment in extracellular vesicles/exosomes suggests that intercellular communication may play a role in the pathogenesis of MASLD [48], while the enrichment in mitochondrial components underscores the central role of mitochondrial dysfunction in the disease. At the MF level, the DEPs’ enrichment closely mirrored the biological processes observed, with oxidoreductase activity and electron transfer activity further emphasizing the importance of oxidative stress in disease mechanisms. The enrichment of protein disulfide isomerase activity, which is associated with the ER stress response, suggests a disruption in the protein quality control system. KEGG pathway analysis revealed that oxidative phosphorylation and protein processing in the ER were the most significantly enriched pathways for DEPs. Downregulation of respiratory chain complex subunits, such as UQCRC1, NDUFS1, and SDHB, within oxidative phosphorylation pathways, impairs mitochondrial electron transport chain function, leading to reduced ATP production. These alterations not only result from lipid accumulation and IR in hepatocytes but also contribute to their progression [49]. Notably, enrichment in neurodegenerative disease pathways, including Parkinson’s disease and Huntington’s disease, suggests that MASLD and neurodegenerative diseases may share a common molecular basis. Both oxidative stress and mitochondrial dysfunction are key contributors to metabolic disorders like MASLD, as well as aging-related neurodegenerative diseases, supporting findings from Jha et al. [50].

The PPI network constructed in this study revealed multiple key hub proteins and functional modules. P4HB, the most connected node in the network, is a critical molecular chaperone in the ER, involved in collagen synthesis and protein folding processes. Recent studies have highlighted abnormal expression of P4HB in metabolic diseases. Gong [51] observed significant differences in P4HB expression in the livers of diabetic gerbils compared to control animals. Wang [52] reported that high glucose induced increased P4HB expression in renal tubular epithelial cells, contributing to their epithelial-mesenchymal transition and fibrosis. Additionally, P4HB-deficient islet β cells exhibit ultrastructural abnormalities, including ER vesiculation, dilation, mitochondrial swelling, and nuclear condensation [53]. Thus, P4HB may influence hepatocyte homeostasis under high glucose conditions by regulating ER function and the unfolded protein response (UPR). Network analysis revealed that P4HB tightly clustered with several protein disulfide isomerases, including CALR, PDIA3, PDIA4, and PDIA6, collectively forming an ER quality control system. This finding aligns with the significant enrichment of the ‘protein processing in the ER’ pathway in KEGG analysis, underscoring the central role of ER stress in the pathogenesis of T2DM combined with MASLD [54]. Another crucial functional module involved subunits of the mitochondrial respiratory chain complex, such as UQCRC1, UQCRC2, NDUFS1, NDUFS2, SDHA, and SDHB, all integral to oxidative phosphorylation. The widespread downregulation of these subunits reflects severe impairment of mitochondrial energy metabolism in T2DM patients with MASLD, consistent with mitochondrial dysfunction observed during the progression of MASLD [55]. Notably, the downregulation of these subunits suggests a potential link to reduced mitochondrial biosynthesis due to abnormal PGC-1α signaling [56]. PPI network analysis not only confirmed the results of pathway enrichment analysis but also highlighted the interconnectedness of these pathways, revealing the molecular characteristics of multi-organelle dysfunction in T2DM combined with MASLD.

XPNPEP3, identified as the most significantly downregulated protein with the largest fold change in this study. Previous research on XPNPEP3 has primarily focused on its association with autosomal recessive tubulointerstitial diseases, such as nephronophthisis-like nephropathy-1 (NPHPL1). Through proteomic analysis, this study is the first to report that XPNPEP3 is significantly downregulated in the plasma of T2DM patients with MASLD, thereby extending its functional implications to metabolic diseases, particularly metabolic liver diseases associated with mitochondrial dysfunction. To further validate the expression changes and diagnostic efficacy of XPNPEP3 in T2DM with MASLD, the study cohort was expanded to include 42 patients with T2DM alone and 42 patients with both T2DM and MASLD. Clinical and biochemical characteristics were compared between the two groups. Patients in the T2DM + MASLD group were younger yet exhibited a longer duration of diabetes compared to those with T2DM alone. This seemingly paradoxical finding highlights the close and complex relationship between early-onset type 2 diabetes (EOT2D, typically defined as diagnosis before age 40) and MASLD. EOT2D is characterized by more rapid β-cell decline and more severe IR, driving accelerated disease progression [57]. Patients are diagnosed at a younger age, so their exposure to hyperglycemia is actually longer. This provides a long-term metabolic disorder environment for the development of MASLD. Recent evidence indicates a higher prevalence of MASLD in EOT2D compared to those with later-onset disease. Furthermore, patients with both EOT2D and MASLD exhibit more significant IR, poorer glycemic control, elevated liver enzyme levels, and more severe dyslipidemia, all of which drive MASLD progression [58]. Thus, our findings likely reveal a more aggressive phenotype characterized by the co-occurrence of EOT2D and MASLD. Additionally, in patients with later-onset type 2 diabetes, the disease pathogenesis may be more attributable to the age-related decline in β-cell function, while insulin resistance might be relatively less severe [59]. This could partially explain why patients in the T2DM group were older but had a shorter duration of diabetes. It is important to acknowledge that these population characteristics may also be influenced by potential selection bias in our study cohort. The BMI, liver damage markers, TG, and TCH levels in the T2DM + MASLD group were significantly higher compared to the T2DM group, consistent with findings from Xing [60] and the clinical phenotype of MASLD. This indicates that T2DM patients with MASLD experience more severe metabolic disorders and liver damage.

In the expanded cohort, plasma XPNPEP3 levels were significantly lower in the T2DM + MASLD group compared to the T2DM group, confirming our earlier proteomic results. While direct evidence explaining the upstream mechanisms of its downregulation is currently lacking, our finding may reveal a potential vicious cycle in the pathogenesis of T2DM comorbid with MASLD. In the context of insulin resistance in T2DM, initial metabolic stress (such as lipid overload and hyperglycemia) may first induce mild mitochondrial stress and dysfunction [61]. The downregulation of the mitochondrial protein XPNPEP3 observed in this study could be a sensitive reflection or maladaptive response to such early mitochondrial impairment. Importantly, as documented in the literature, the function of XPNPEP3 is essential for maintaining respiratory chain complex activity and oxidative phosphorylation efficiency. Consequently, once XPNPEP3 expression or function is compromised, it would further exacerbate mitochondrial dysfunction, leading to impaired fatty acid β-oxidation and excessive ROS production, thereby promoting hepatic fat accumulation. The resulting oxidative stress and lipotoxicity may further suppress the expression of mitochondrial proteins, including XPNPEP3, thereby creating a vicious cycle of disease progression. Thus, XPNPEP3 not only protects mitochondrial function, but its reduction may also be a key event that triggers the collapse of mitochondrial homeostasis under metabolic stress.

Subsequent analysis of the correlation between plasma XPNPEP3 levels and various clinical and biochemical indicators revealed that XPNPEP3 was negatively correlated with the duration of diabetes. This suggests that prolonged IR, metabolic disorders, and metabolic stress may accelerate the decline in its expression, reflecting how chronic mitochondrial overload can damage mitochondria and induce dysfunction [62]. Additionally, XPNPEP3 was negatively correlated with ALT, AST, ALP, GGT, and TG, implying that it may play a role in reducing liver lipid accumulation and damage, as well as mitigating metabolic disorders. It may also serve as a dynamic indicator for tracking disease progression. Notably, in the T2DM + MASLD group, XPNPEP3 levels showed a negative correlation with serum Cre levels, suggesting a potential link to early kidney function impairment. This finding is particularly relevant in the context of the established ‘liver-kidney axis’ in metabolic diseases. Epidemiological studies consistently show that patients with MASLD have a significantly higher prevalence of chronic kidney disease (CKD) [63], with the severity of MASLD being positively associated with the risk of CKD [64]. Dysfunctional liver metabolism in MASLD can contribute to kidney injury through several pathways. This includes the release of inflammatory signaling molecules (such as TNF-α and IL-6) [65], changes in bile acid metabolism [66], and the systemic release of lipotoxic substances [67]. Our finding aligns with previous conclusions that XPNPEP3 mutations are associated with renal tubulointerstitial dysfunction. In mice, loss of XPNPEP3 results in reduced mitochondrial complex I activity, primary cilia elongation, and a predisposition to renal tubular dilatation and fibrosis under stress [26]. Therefore, XPNPEP3 may represent a shared molecular link in the metabolic dysfunction connecting the liver and the kidneys.

Univariate logistic regression analysis indicated that a longer diabetes duration was significantly associated with a higher likelihood of MASLD in T2DM patients. Several studies have established that T2DM and IR are key risk factors for the development of MASLD [68–69]. As diabetes duration lengthens, the degree of IR intensifies, and chronic glucolipotoxicity accelerates the progression of MASLD. Additionally, higher BMI, liver function indicators (ALB, ALT, AST), and lipid levels were all significantly associated with MASLD. Similar findings were observed in a cross-sectional study in Egypt by Semeya et al. [70]. Importantly, considering the difference in diabetes duration between the discovery cohort (median < 1 month) and the validation cohort (median 42.0–96.0 months), and to evaluate the independent association between XPNPEP3 and MASLD, we performed multivariable logistic regression analysis in the validation cohort with adjustment for diabetes duration and other covariates. In the multivariable analysis adjusted for age, gender, BMI, and diabetes duration, lower plasma XPNPEP3 levels remained independently associated with MASLD in T2DM patients. ROC curve analysis showed that the AUC for XPNPEP3 alone was 0.714 for distinguishing MASLD. When combined with BMI and diabetes duration, the AUC increased to 0.779, indicating a significant improvement in diagnostic efficiency. Therefore, XPNPEP3 not only shows substantial independent value but also enhances the identification of MASLD in T2DM patients when combined with other clinical indicators. Currently, various non-invasive biomarkers and scores are used in the clinical assessment of MASLD. These include simple serum markers (such as ALT, AST), combined scores (such as FIB-4, APRI), and tests like the ELF^™^ test [71]. While these markers are useful for detecting advanced fibrosis, many lack optimal sensitivity for early-stage MASLD [72] or show limited specificity in populations with complex metabolic disorders such as T2DM [73]. In this context, our findings on XPNPEP3 suggest potential complementary value. Unlike conventional liver enzymes that reflect general liver cell injury, XPNPEP3 is a mitochondrial protein. Its reduced expression may more directly reflect mitochondrial dysfunction, which is a key feature of both T2DM and MASLD. Furthermore, in our T2DM cohort rigorously controlled for key confounders, XPNPEP3 showed a strong and independent association with MASLD. Its unique biological basis could aid in risk stratification within metabolically complex T2DM populations or serve as a component of future multi-parameter models for early MASLD detection. Future studies are needed to validate its added value to combined scores in larger cohorts to move toward clinical use.

The evidence presented in this study collectively highlights the critical role of XPNPEP3 in T2DM with MASLD. Downregulation of XPNPEP3 was observed in plasma proteomics and subsequently validated by plasma ELISA. Notably, this study directly confirmed a significant reduction in XPNPEP3 protein expression in the liver, the target organ of disease, through immunohistochemical analysis of liver tissue. This finding establishes a direct correlation between biomarker changes in peripheral blood and altered molecular pathology in target organs, greatly enhancing the credibility of XPNPEP3 as a potential biomarker. Combined with the known molecular function of XPNPEP3 and the significant enrichment of the ‘oxidative phosphorylation’ pathway identified in our bioinformatics analysis, XPNPEP3 reduction in hepatocytes may directly disrupt mitochondrial peptide processing and function, impairing mitochondrial function and inducing metabolic stress. This likely promotes liver lipid accumulation and may lead to the leakage of mitochondrial proteins and other contents into the bloodstream [74]. Thus, decreased XPNPEP3 levels in peripheral blood in T2DM patients with MASLD may serve as a direct reflection of reduced liver-derived tissue expression, providing strong evidence for XPNPEP3 as a molecular marker of liver mitochondrial health.

However, this study has some limitations. First, the sample size was calculated for the primary analysis, leaving the subgroup correlation analyses underpowered and exploratory, and the single-center design may limit generalizability. Future studies incorporating multicenter data are warranted to validate our findings. Second, the cross-sectional design does not establish a causal relationship between low XPNPEP3 levels and the development of MASLD. A longitudinal cohort study would be needed to clarify the relationship between XPNPEP3 dynamics and disease progression. Third, our study was conducted exclusively in patients with T2DM, a population at high risk for MASLD. While this design is appropriate for addressing the clinically relevant question of identifying MASLD within this specific group, it does not allow us to determine whether our findings can be generalized to non-diabetic individuals or to the general population. Future studies including healthy controls and non-diabetic MASLD patients are warranted to further validate the role of XPNPEP3 in broader populations. Finally, the specific functional mechanism of XPNPEP3 in T2DM with MASLD has not been validated through *in vitro* or *in vivo* experiments, and it remains unclear whether XPNPEP3 directly regulates mitochondrial function. Therefore, future studies should incorporate *in vitro* and *in vivo* models to explore the molecular mechanisms of XPNPEP3 in the disease, providing a more solid scientific basis for its clinical application.

## Conclusion

This study used directDIA mass spectrometry to analyze protein expression profiles in the plasma of T2DM patients with and without MASLD, identifying 176 DEPs, of which 16 were upregulated and 160 were downregulated. Bioinformatics analysis revealed that these DEPs were significantly associated with oxidative phosphorylation and protein processing in the ER. Additionally, this study is the first to report that XPNPEP3 levels in plasma and liver tissues are significantly lower in T2DM patients with MASLD compared to those without MASLD. XPNPEP3 was identified as a biomarker for MASLD in T2DM patients, and the combined diagnostic model incorporating XPNPEP3 with BMI and diabetes duration further enhanced diagnostic efficiency. In conclusion, XPNPEP3, as a novel biomarker related to mitochondrial function, offers new insights into the mechanisms of T2DM and MASLD comorbidity and lays a theoretical foundation for the development of non-invasive diagnostic tools and novel therapeutic targets.

## Supplementary Material

Supplemental Material

Table_SII_Differentially_Expressed_Proteins_List.docx

Table SIII Protein Protein Interaction Network.docx

FigureS1.jpg

Table SI Clinical Characteristics of 24 T2DM Patients With or Without MASLD for IHC.docx

## Data Availability

The datasets used and/or analyzed during the current study are available from the corresponding author on reasonable request.
