# Supplementary material for: Plasma proteome profiling identifies XPNPEP3 as a novel biomarker associated with metabolic dysfunction-associated steatotic liver disease in patients with type 2 diabetes mellitus
Source: Ann Med. 2026 Apr 13;58(1):2654911. doi: 10.1080/07853890.2026.2654911 (PMC13078656; doi:10.1080/07853890.2026.2654911)
Supplement: Supplemental Material [file IANN_A_2654911_SM3987.docx]

Figure S1 Cluster diagram illustrating the DEPs in T2DM patients with and without MASLD. Abbreviations: T2DM, Type 2 Diabetes Mellitus; MASLD, Metabolic Dysfunction-associated Steatotic Liver Disease; DEPs, Differentially Expressed Proteins.
