# Supplementary material for: Plasma proteome profiling identifies XPNPEP3 as a novel biomarker associated with metabolic dysfunction-associated steatotic liver disease in patients with type 2 diabetes mellitus
Source: Ann Med. 2026 Apr 13;58(1):2654911. doi: 10.1080/07853890.2026.2654911 (PMC13078656; doi:10.1080/07853890.2026.2654911)
Supplement: Table_SII_Differentially_Expressed_Proteins_List.docx [file IANN_A_2654911_SM6032.docx]

**Table SII** Differentially Expressed Proteins List

| **Gene Name** | **Accession** | **Mean T2DM** | **Mean T2DM+MASLD** | ***P*-value** | **Q-value** | ***P*-value-chitest** | **Fold change** | **Log-fold change** |
| --- | --- | --- | --- | --- | --- | --- | --- | --- |
| XPNPEP3 | Q9NQH7 | 237297.11 | 2534.3212 | 1.01129E-05 | 0.026456902 | 5.22787E-05 | 0.01067995 | -6.548951337 |
| HLA-G | P17693 | 103817.99 | 2115.1484 | 0.000634119 | 0.482628134 | 0.00026073 | 0.020373621 | -5.617153764 |
| BAG5 | Q9UL15 | 3123.4142 | 136.568 | 0.009338814 | 0.652605148 | 0.003414791 | 0.043723948 | -4.515432517 |
| PDGFRL | Q15198 | 2632.809 | 136.568 | 0.033505587 | 0.664909498 | 0.009823275 | 0.051871594 | -4.268911481 |
| STUM | Q69YW2 | 2545.256 | 136.568 | 0.050224767 | 0.666526072 | 0.009823275 | 0.053655899 | -4.220119386 |
| ENTPD5 | O75356 | 1952.573 | 136.568 | 0.034417972 | 0.665037944 | 0.009823275 | 0.069942583 | -3.837685104 |
| CPVL | Q9H3G5 | 1706.608 | 136.568 | 0.024713207 | 0.663190415 | 0.009823275 | 0.080023063 | -3.643440333 |
| IGHV1-69-2 | A0A0G2JMI3 | 11295.8172 | 1037.9692 | 0.015186446 | 0.659116645 | 0.019076322 | 0.091889695 | -3.443953107 |
| PCYOX1L | Q8NBM8 | 5761.7242 | 532.7032 | 0.014428227 | 0.658565174 | 0.019076322 | 0.092455519 | -3.435096751 |
| ARMC8 | Q8IUR7 | 31391.2644 | 2994.7244 | 0.052737864 | 0.666680861 | 0.024618761 | 0.095399929 | -3.389867995 |
| DYRK1A | Q13627 | 4227.07 | 431.8172 | 0.029270892 | 0.664209378 | 0.050961937 | 0.102155204 | -3.291165389 |
| MRPL12 | P52815 | 6141.662 | 723.4792 | 0.0431892 | 0.665997466 | 0.050961937 | 0.117798602 | -3.085605672 |
| NIPSNAP3A | Q9UFN0 | 10644.2804 | 1325.3934 | 0.009525759 | 0.652933907 | 0.024618761 | 0.124516957 | -3.005585875 |
| SLC35A4 | L0R6Q1 | 5258.3034 | 782.3342 | 0.021347599 | 0.662161869 | 0.006169899 | 0.148780726 | -2.748740447 |
| SLC25A12 | O75746 | 888.8183 | 136.568 | 0.096625715 | 0.668089233 | 0.025347319 | 0.153651202 | -2.702269043 |
| NDUFS2 | O75306 | 5225.2402 | 815.9902 | 0.02566892 | 0.663433782 | 0.019076322 | 0.156163194 | -2.678873628 |
| SCPEP1 | Q9HB40 | 2898.4994 | 467.5082 | 0.02514148 | 0.663301738 | 0.024618761 | 0.161293185 | -2.632242612 |
| TMCC2 | O75069 | 7652.024 | 1262.1694 | 0.041356925 | 0.665830457 | 0.159598942 | 0.164945823 | -2.599935846 |
| NLRX1 | Q86UT6 | 2763.485 | 457.3812 | 0.036231681 | 0.665274197 | 0.050961937 | 0.165508841 | -2.59501981 |
| GUCY1A1 | Q02108 | 4587.7144 | 778.7299 | 0.009588725 | 0.653041824 | 0.024618761 | 0.169742454 | -2.558580656 |
| DIABLO | Q9NR28 | 1828.517 | 313.8702 | 0.034563716 | 0.665057838 | 0.050961937 | 0.171652875 | -2.542434074 |
| HSD17B12 | Q53GQ0 | 5032.025 | 876.7752 | 0.041991744 | 0.66588996 | 0.050961937 | 0.174239039 | -2.520860193 |
| PCCB | P05166 | 2793.465 | 498.8532 | 0.042708491 | 0.665955029 | 0.050961937 | 0.178578647 | -2.48536851 |
| TGOLN2 | O43493 | 6229.2312 | 1121.7002 | 0.059194661 | 0.66701854 | 0.019076322 | 0.180070407 | -2.473366985 |
| LRIG1 | Q96JA1 | 21458.104 | 3895.4014 | 0.047982448 | 0.666374343 | 0.159598942 | 0.181535209 | -2.461678705 |
| SLC25A11 | Q02978 | 4827.9432 | 934.9314 | 0.031254559 | 0.664560773 | 0.067889155 | 0.193650041 | -2.368476286 |
| TUBB6 | Q9BUF5 | 4463.2314 | 882.3678 | 0.007089544 | 0.647335908 | 0.07363827 | 0.197697077 | -2.338636557 |
| TXNDC5 | Q8NBS9 | 12336.3258 | 2518.8254 | 0.001014389 | 0.539100425 | 0.001653695 | 0.204179546 | -2.292089746 |
| HINT2 | Q9BX68 | 12616.1636 | 2607.5516 | 0.015727471 | 0.659478139 | 0.024618761 | 0.206683401 | -2.274505566 |
| RAB3GAP2 | Q9H2M9 | 4133.0862 | 865.9342 | 0.043312364 | 0.666008188 | 0.019076322 | 0.209512737 | -2.254890145 |
| SDHA | P31040 | 7586.3356 | 1634.9766 | 0.034325857 | 0.665025284 | 0.024618761 | 0.215515986 | -2.214133206 |
| TRPC6 | Q9Y210 | 4286.0252 | 929.1272 | 0.034484061 | 0.665046986 | 0.019076322 | 0.21678062 | -2.205692308 |
| PDHA1 | P08559 | 6059.3064 | 1349.7084 | 0.033412732 | 0.664896036 | 0.024618761 | 0.222749653 | -2.166504908 |
| CTDSPL | O15194 | 4868.6384 | 1126.2314 | 0.01159897 | 0.655884143 | 0.024618761 | 0.23132369 | -2.112015074 |
| PFDN2 | Q9UHV9 | 4206.3812 | 980.9296 | 0.034986297 | 0.665114591 | 0.177529852 | 0.233200358 | -2.100358094 |
| IVD | P26440 | 8043.8476 | 1895.8796 | 0.014505946 | 0.658624308 | 0.024618761 | 0.235693128 | -2.085018401 |

| GOT2 | P00505 | 38880.9236 | 9366.6076 | 0.002794885 | 0.615623632 | 0.024618761 | 0.240904967 | -2.053463958 |
| --- | --- | --- | --- | --- | --- | --- | --- | --- |
| SDHB | P21912 | 12082.1116 | 2946.6636 | 0.088097445 | 0.667925097 | 0.024618761 | 0.243886474 | -2.035718345 |
| VAPB | O95292 | 4472.1722 | 1094.5602 | 0.047545077 | 0.666343088 | 0.019076322 | 0.244749118 | -2.030624436 |
| MYCBP | Q99417 | 4796.4944 | 1182.1664 | 0.032556967 | 0.664768374 | 0.024618761 | 0.246464668 | -2.020547251 |
| C2orf88 | Q9BSF0 | 6899.4514 | 1730.9634 | 0.037224113 | 0.665393789 | 0.024618761 | 0.250884208 | - 1.994906432 |
| SNAP29 | O95721 | 2677.6244 | 676.8844 | 0.04609781 | 0.66623546 | 0.024618761 | 0.252792886 | - 1.98397223 |
| ERGIC1 | Q969X5 | 5988.4506 | 1540.2266 | 0.00909239 | 0.652151676 | 0.024618761 | 0.257199517 | - 1.959040163 |
| ACADSB | P45954 | 3813.2082 | 987.9922 | 0.088575863 | 0.66793514 | 0.019076322 | 0.259097366 | - 1.948433747 |
| ASAH1 | Q13510 | 10630.0256 | 2761.8306 | 0.027150341 | 0.663777461 | 0.024618761 | 0.259814106 | - 1.944448333 |
| ZZEF1 | O43149 | 5846.1914 | 1522.4854 | 0.035441252 | 0.665174188 | 0.024618761 | 0.260423461 | - 1.941068669 |
| TPP1 | O14773 | 15879.4764 | 4137.5548 | 0.035540235 | 0.665186954 | 0.177529852 | 0.260559901 | - 1.940313018 |
| ABHD11 | Q8NFV4 | 14850.4814 | 3889.3286 | 0.044577222 | 0.666114893 | 0.07363827 | 0.26189916 | - 1.932916664 |
| UBQLN1 | Q9UMX0 | 2267.6432 | 594.8802 | 0.065503171 | 0.667284408 | 0.019076322 | 0.262334127 | - 1.930522593 |
| PFDN5 | Q99471 | 3074.8882 | 806.7824 | 0.032660311 | 0.664784144 | 0.067889155 | 0.2623778 | - 1.930282439 |
| TPST2 | O60704 | 8641.5204 | 2306.4312 | 0.047544097 | 0.666343017 | 0.639207431 | 0.266901088 | - 1.905622906 |
| RBPMS2 | Q6ZRY4 | 4624.5646 | 1243.7066 | 0.033503078 | 0.664909135 | 0.024618761 | 0.268934853 | - 1.89467136 |
| EGF | P01133 | 3800.0554 | 1023.2354 | 0.072515106 | 0.667525822 | 0.024618761 | 0.269268548 | - 1.892882369 |
| SLA2 | Q9H6Q3 | 4133.6042 | 1123.9044 | 0.039326918 | 0.665627367 | 0.067889155 | 0.271894537 | - 1.87888093 |
| TMED7 | Q9Y3B3 | 6762.4374 | 1841.9856 | 0.037373375 | 0.66541123 | 0.07363827 | 0.272384865 | - 1.876281551 |
| DECR1 | Q16698 | 23288.6 | 6381.024 | 0.005982654 | 0.643345641 | 0.009823275 | 0.27399775 | - 1.867764049 |
| AP1G1 | O43747 | 13731.3568 | 3814.3402 | 0.021575224 | 0.662241436 | 0.12133525 | 0.277783198 | - 1.847968758 |
| NDUFV2 | P19404 | 9958.1938 | 2806.2116 | 0.005225581 | 0.639686683 | 0.006169899 | 0.281799256 | - 1.827260294 |
| ACOT9 | Q9Y305 | 5335.7026 | 1517.1278 | 0.039993115 | 0.665696275 | 0.067889155 | 0.28433515 | - 1.814335636 |
| GRAP2 | O75791 | 10979.8552 | 3138.9178 | 0.04629278 | 0.66625035 | 0.37109337 | 0.285879708 | - 1.806519874 |
| IMPDH2 | P12268 | 7113.4644 | 2047.4228 | 0.031954097 | 0.664674366 | 0.177529852 | 0.287823581 | - 1.796743298 |
| GATD3B | A0A0B4J2D5 | 4462.7512 | 1318.9521 | 0.044498912 | 0.666108462 | 0.177529852 | 0.295546859 | - 1.758541206 |
| TSN | Q15631 | 6206.9358 | 1836.2686 | 0.029570837 | 0.664265512 | 0.006169899 | 0.295841404 | - 1.75710412 |
| HSPA7 | P48741 | 63194.3704 | 19583.6244 | 0.048304308 | 0.666396983 | 0.024618761 | 0.309895079 | - 1.69014825 |
| RHOF | Q9HBH0 | 4864.7816 | 1514.1334 | 0.063116931 | 0.667190067 | 0.007290358 | 0.311243859 | - 1.683882721 |
| MAOB | P27338 | 22280.0198 | 7116.9022 | 0.024176201 | 0.663045311 | 0.12133525 | 0.319429797 | - 1.646429199 |
| SFXN3 | Q9BWM7 | 12922.4204 | 4134.4976 | 0.0307832 | 0.664481344 | 0.07363827 | 0.319947616 | - 1.644092378 |
| REG4 | Q9BYZ8 | 3707.6872 | 1187.1812 | 0.131210251 | 0.668536552 | 0.019076322 | 0.320194541 | - 1.642979385 |
| IGFBP1 | P08833 | 659147.37 | 215851.73 | 0.030497224 | 0.664431967 |  | 0.327471124 | - 1.610560399 |
| ACADVL | P49748 | 28546.166 | 9373.8882 | 0.01366358 | 0.657948139 | 0.025347319 | 0.328376434 | - 1.606577499 |
| RTN2 | O75298 | 25019.2436 | 8248.2018 | 0.040613978 | 0.665758471 | 0.067889155 | 0.329674307 | - 1.600886638 |
| SMTN | P53814 | 15167.3196 | 5009.0818 | 0.026767764 | 0.663692316 | 0.067889155 | 0.330254912 | - 1.598348075 |
| GARS1 | P41250 | 7814.2378 | 2601.3838 | 0.004787205 | 0.63706478 | 0.019076322 | 0.332903076 | - 1.586825894 |
| UQCRQ | O14949 | 15006.0846 | 5081.9008 | 0.02001833 | 0.661661521 | 0.067889155 | 0.338656014 | - 1.562107478 |
| UBA7 | P41226 | 7068.2056 | 2393.9324 | 0.058598895 | 0.666990485 | 0.007290358 | 0.338690261 | - 1.561961592 |
| SHMT2 | P34897 | 10369.1558 | 3521.0498 | 0.018109453 | 0.660816248 | 0.019076322 | 0.339569572 | - 1.558220905 |

| MAP3K5 | Q99683 | 7704.9984 | 2647.7144 | 0.074895989 | 0.667597547 | 0.024618761 | 0.343635944 | - 1.541047145 |
| --- | --- | --- | --- | --- | --- | --- | --- | --- |
| ITPR1 | Q14643 | 8097.5394 | 2813.4708 | 0.039072155 | 0.665600398 | 0.177529852 | 0.347447621 | - 1.525132592 |
| MMP19 | Q99542 | 6176.6914 | 2158.2002 | 0.045134217 | 0.666159995 | 0.639207431 | 0.349410398 | - 1.517005554 |
| NDUFA4 | O00483 | 26257.0036 | 9293.7276 | 0.060418937 | 0.66707446 | 0.024618761 | 0.35395233 | - 1.498373023 |
| B4GALT1 | P15291 | 7060.5228 | 2511.1696 | 0.046092062 | 0.66623502 | 0.006169899 | 0.355663408 | - 1.491415544 |
| ARPC5L | Q9BPX5 | 6179.3546 | 2203.723 | 0.01674578 | 0.660096097 | 0.159598942 | 0.356626726 | - 1.487513271 |
| UQCRC2 | P22695 | 15219.4156 | 5436.888 | 0.026468046 | 0.663623908 | 0.159598942 | 0.35723369 | - 1.485059949 |
| AK2 | P54819 | 16308.2536 | 5919.6128 | 0.045322205 | 0.666174968 | 0.067889155 | 0.362982631 | - 1.462027579 |
| COPB1 | P53618 | 8322.3806 | 3022.6238 | 0.032080947 | 0.664694437 | 0.067889155 | 0.36319221 | - 1.461194837 |
| STON2 | Q8WXE9 | 3011.4284 | 1095.5276 | 0.044882816 | 0.666139776 | 0.07363827 | 0.363790021 | - 1.458822127 |
| NDUFS1 | P28331 | 11366.5718 | 4145.536 | 0.020312255 | 0.661777729 | 0.050961937 | 0.364712956 | - 1.455166645 |
| SSR4 | P51571 | 8619.0136 | 3169.517 | 0.040044363 | 0.665701481 | 0.159598942 | 0.367735468 | - 1.443259764 |
| LRPPRC | P42704 | 9575.8236 | 3522.6186 | 0.045699648 | 0.666204661 | 0.024618761 | 0.367865862 | - 1.442748296 |
| PSMD4 | P55036 | 13074.1696 | 4847.113 | 0.036298286 | 0.665282426 | 0.159598942 | 0.370739645 | - 1.431521696 |
| ATP5MG | O75964 | 40268.241 | 14955.0394 | 0.018536118 | 0.661020089 | 0.060289174 | 0.371385465 | - 1.429010742 |
| PEAR1 | Q5VY43 | 20584.6788 | 7658.387 | 0.02877809 | 0.664114633 | 0.050961937 | 0.372043065 | - 1.426458467 |
| COX6C | P09669 | 32393.471 | 12063.9964 | 0.043716175 | 0.666042921 | 0.060289174 | 0.372420615 | - 1.42499516 |
| UQCRC1 | P31930 | 14682.7058 | 5474.6208 | 0.018097569 | 0.660810434 | 0.019076322 | 0.372861847 | - 1.423286915 |
| ACTBL2 | Q562R1 | 41737.9136 | 15781.614 | 0.040000549 | 0.665697031 | 0.159598942 | 0.378112192 | - 1.403113726 |
| PSMD5 | Q16401 | 27567.6668 | 10477.1108 | 0.063275661 | 0.667196562 | 0.019076322 | 0.38005069 | - 1.39573624 |
| VDAC1 | P21796 | 18828.938 | 7294.4642 | 0.044003429 | 0.666067243 | 0.025347319 | 0.387407096 | - 1.368077714 |
| CAD | P27708 | 6074.2696 | 2378.8648 | 0.026651301 | 0.663665916 | 0.067889155 | 0.391629769 | - 1.352437662 |
| CDH23 | Q9H251 | 4610.2306 | 1820.9986 | 0.079087019 | 0.667713344 | 0.024618761 | 0.394990784 | - 1.340109102 |
| ARL15 | Q9NXU5 | 11128.0478 | 4418.1718 | 0.027351735 | 0.663821333 | 0.019076322 | 0.397030268 | - 1.332679098 |
| NID2 | Q14112 | 9262.981 | 3726.1002 | 0.001222886 | 0.557651845 | 0.025347319 | 0.402257135 | - 1.313810084 |
| HADHB | P55084 | 27547.269 | 11111.2042 | 0.035440623 | 0.665174107 | 0.025347319 | 0.403350481 | - 1.309894119 |
| PPP6C | O00743 | 5565.8658 | 2269.0248 | 0.031620166 | 0.664620763 | 0.019076322 | 0.407667896 | - 1.294533746 |
| PRDX4 | Q13162 | 23979.866 | 9869.674 | 0.029595258 | 0.664270033 | 0.009823275 | 0.411581699 | - 1.280749259 |
| CISD2 | Q8N5K1 | 20393.792 | 8466.1572 | 0.012304716 | 0.656666068 | 0.025347319 | 0.415134037 | - 1.268350871 |
| DCTN1 | Q14203 | 11347.9326 | 4714.1334 | 0.034030513 | 0.664984233 | 0.605576616 | 0.415417818 | - 1.267364998 |
| PDIA6 | Q15084 | 26291.4668 | 11271.5314 | 0.017531123 | 0.660524326 | 0.263552477 | 0.428714437 | - 1.221911092 |
| PRDX3 | P30048 | 27716.445 | 11892.7568 | 0.025965862 | 0.663505784 | 0.304901788 | 0.42908666 | - 1.220659047 |
| TMED10 | P49755 | 24516.5538 | 10668.9762 | 0.041676633 | 0.66586065 | 0.12133525 | 0.435174384 | - 1.200334458 |
| LCK | P06239 | 13388.563 | 5833.4698 | 0.029503074 | 0.664252929 | 0.003414791 | 0.435705445 | - 1.198574952 |
| HARS1 | P12081 | 7648.441 | 3335.092 | 0.010062546 | 0.653811619 | 0.009823275 | 0.436048601 | - 1.19743915 |
| ERP29 | P30040 | 21034.0368 | 9238.684 | 0.024858226 | 0.663228536 | 0.050961937 | 0.439225437 | - 1.186966488 |
| PDLIM7 | Q9NR12 | 48133.2668 | 21279.9104 | 0.048898636 | 0.666438011 | 0.263552477 | 0.442104013 | - 1.177542266 |
| GLRX | P35754 | 7462.5268 | 3307.636 | 0.029237336 | 0.664203027 | 0.050961937 | 0.443232713 | - 1.173863731 |
| GPD2 | P43304 | 28970.1868 | 12979.5672 | 0.043620813 | 0.666034777 | 0.12133525 | 0.448031878 | - 1.158326709 |
| RFTN1 | Q14699 | 2916.3206 | 1315.9336 | 0.102002021 | 0.668178635 | 0.024618761 | 0.451230773 | - 1.148062634 |

| GP9 | P14770 | 294797.79 | 135294.8 | 0.031021938 | 0.664521873 |  | 0.458941025 | - 1.123619318 |
| --- | --- | --- | --- | --- | --- | --- | --- | --- |
| COPA | P53621 | 21449.97 | 9996.283 | 0.035145963 | 0.665135681 | 0.009823275 | 0.466027831 | - 1.101511979 |
| IMMT | Q16891 | 10963.6198 | 5205.1402 | 0.028817436 | 0.664122315 | 0.12133525 | 0.474764749 | - 1.074715276 |
| MTTP | P55157 | 10873.466 | 5195.8982 | 0.02012537 | 0.661704229 | 0.025347319 | 0.477851147 | - 1.065366813 |
| CYB5R3 | P00387 | 92502.667 | 44505.2 | 0.048527147 | 0.666412483 |  | 0.481123425 | - 1.055521051 |
| STX4 | Q12846 | 13143.8268 | 6388.4708 | 0.050272094 | 0.666529129 | 0.019076322 | 0.486043441 | - 1.040842833 |
| ITGAV | P06756 | 6244.629 | 3035.5242 | 0.006427749 | 0.645109294 | 0.025347319 | 0.486101608 | - 1.040670188 |
| ATP6V1H | Q9UI12 | 11156.9506 | 5426.8112 | 0.354606619 | 0.682265357 | 0.001653695 | 0.486406312 | - 1.039766143 |
| EEF1B2 | P24534 | 50145.52 | 24533.6136 | 0.034561909 | 0.665057593 | 0.136037128 | 0.489248364 | - 1.03136107 |
| ARPC4 | P59998 | 140532.64 | 68783.93 | 0.025378323 | 0.663361704 |  | 0.489451632 | - 1.030761796 |
| VAPA | Q9P0L0 | 22003.6668 | 10822.3016 | 0.041305578 | 0.665825565 | 0.531167837 | 0.491840824 | - 1.023736609 |
| DYNLL1 | P63167 | 8947.331 | 4445.4014 | 0.017477845 | 0.660496474 | 0.060289174 | 0.496841058 | - 1.009143695 |
| TMEM40 | Q8WWA1 | 38526.3868 | 19229.1908 | 0.105776931 | 0.668235989 | 0.019076322 | 0.49911742 | - 1.002548836 |
| NCK2 | O43639 | 13995.904 | 7092.5696 | 0.007482106 | 0.648476491 | 0.136037128 | 0.506760378 | -0.980624366 |
| NDST1 | P52848 | 14157.876 | 7177.3922 | 0.130066067 | 0.668525551 | 0.025347319 | 0.506954023 | -0.980073183 |
| NONO | Q15233 | 24190.7168 | 12706.0672 | 0.035898073 | 0.665232521 | 0.12133525 | 0.525245585 | -0.928935964 |
| PHB2 | Q99623 | 14474.179 | 7621.0382 | 0.056708381 | 0.666897576 | 0.025347319 | 0.526526458 | -0.925422067 |
| UGGT1 | Q9NYU2 | 15859.272 | 8390.2912 | 0.024404503 | 0.663107773 | 0.025347319 | 0.529046428 | -0.91853376 |
| DNAJB11 | Q9UBS4 | 12281.345 | 6605.6126 | 0.007976695 | 0.649758465 | 0.136037128 | 0.537857425 | -0.8947043 |
| NME2 | P22392 | 19462.42 | 10528.3708 | 0.006065621 | 0.643693253 | 0.304901788 | 0.540958976 | -0.886408904 |
| ITLN1 | Q8WWA0 | 13183.484 | 7278.6564 | 0.025977175 | 0.663508495 | 0.060289174 | 0.552104163 | -0.856987615 |
| ACTN4 | O43707 | 31913.51 | 17974.926 | 0.047757903 | 0.666358368 |  | 0.563238766 | -0.82818146 |
| CSNK2A1 | P68400 | 19844.1368 | 11405.2808 | 0.159080911 | 0.668755726 | 0.019076322 | 0.574743105 | -0.799010841 |
| MAPK14 | Q16539 | 35497.1 | 20408.2472 | 0.097131281 | 0.668098061 | 0.025347319 | 0.574927169 | -0.798548887 |
| ACTR2 | P61160 | 81608 | 47046.43 | 0.049831188 | 0.666500423 |  | 0.576492868 | -0.794625334 |
| PDIA3 | P30101 | 74204.94 | 43398.11 | 0.032120426 | 0.664700652 |  | 0.584841252 | -0.773883019 |
| ARPC5 | O15511 | 75688.59 | 44374.08 | 0.040509 | 0.665748088 |  | 0.586271722 | -0.770358623 |
| YWHAQ | P27348 | 12834.533 | 7567.3868 | 0.030565114 | 0.664443772 | 0.304901788 | 0.589611387 | -0.762163709 |
| GANAB | Q14697 | 50219.53 | 29661.84 | 0.014577795 | 0.658678425 |  | 0.590643521 | -0.759640432 |
| P4HB | P07237 | 42471.29 | 25449.31 | 0.012536493 | 0.656904029 |  | 0.59921208 | -0.738861388 |
| DNM2 | P50570 | 24332.375 | 14656.793 | 0.046756474 | 0.666285265 |  | 0.602357682 | -0.731307677 |
| MPST | P25325 | 16646.25 | 10036.957 | 0.046100312 | 0.666235652 |  | 0.602956041 | -0.729875269 |
| USP5 | P45974 | 6617.946 | 4060.3202 | 0.137855291 | 0.66859684 | 0.025347319 | 0.613531782 | -0.704790016 |
| PYGB | P11216 | 33202.96 | 20545.29 | 0.011924546 | 0.656256129 |  | 0.618778868 | -0.692504167 |
| GPNMB | Q14956 | 32134.93 | 19972.989 | 0.043502069 | 0.666024585 |  | 0.621535164 | -0.686092078 |
| CALR | P27797 | 60798.81 | 38035.74 | 0.0290543 | 0.664168129 |  | 0.625600073 | -0.676687413 |
| NID1 | P14543 | 24052.492 | 15625.962 | 0.033109867 | 0.664851604 |  | 0.649660833 | -0.622241364 |
| ASPH | Q12797 | 23034.4 | 14986.6568 | 0.034068525 | 0.664989556 | 0.304901788 | 0.650620672 | -0.620111434 |
| ERAP1 | Q9NZ08 | 15345.942 | 10282.9548 | 0.041195048 | 0.665814992 | 0.304901788 | 0.67007648 | -0.577602326 |
| PDIA4 | P13667 | 36091.54 | 24628.48 | 0.018114029 | 0.660818484 |  | 0.68238928 | -0.551333112 |

| TM9SF2 | Q99805 | 9562.8348 | 6644.1868 | 0.441572619 | 0.707235018 | 0.019076322 | 0.694792594 | -0.525345718 |
| --- | --- | --- | --- | --- | --- | --- | --- | --- |
| HYOU1 | Q9Y4L1 | 37605.3 | 26166.72 | 0.01796303 | 0.660744091 |  | 0.695825322 | -0.523202912 |
| DDAH2 | O95865 | 5819.838 | 4229.057 | 0.317652129 | 0.677258784 | 0.009823275 | 0.726662323 | -0.46064299 |
| RGS10 | O43665 | 13047.54 | 10330.7302 | 0.549928004 | 0.733880748 | 0.025347319 | 0.79177609 | -0.336835594 |
| KRT83 | P78385 | 4629.9428 | 7966.2678 | 0.249791425 | 0.672966888 | 0.019076322 | 1.720597455 | 0.782909609 |
| DEFA4 | P12838 | 1578.0074 | 2741.0014 | 0.362929414 | 0.685081735 | 0.024618761 | 1.737001614 | 0.796599094 |
| SRSF5 | Q13243 | 10631.4596 | 18736.4278 | 0.039637152 | 0.665659742 | 0.531167837 | 1.762357052 | 0.817506243 |
| CES1 | P23141 | 348810.58 | 627481.4 | 0.006718415 | 0.646139358 |  | 1.798917338 | 0.847128895 |
| IFIT5 | Q13325 | 3120.6668 | 6077.9938 | 0.13462239 | 0.668568251 | 0.019076322 | 1.947658686 | 0.961740877 |
| IGLV10-54 | A0A075B6I4 | 10814.4298 | 24299.2508 | 0.118914595 | 0.668407266 | 0.019076322 | 2.246928525 | 1.167954233 |
| ALYREF | Q86V81 | 43729.2036 | 108166.43 | 0.037789167 | 0.665459092 | 0.136037128 | 2.473551336 | 1.306583842 |
| ASGR2 | P07307 | 3012.1906 | 8303.887 | 0.009160445 | 0.652279286 | 0.001032014 | 2.756760147 | 1.46297375 |
| NDRG1 | Q92597 | 14691.2808 | 52235.8268 | 0.002602941 | 0.61197415 | 0.019076322 | 3.555566564 | 1.830079465 |
| IGLV3- 12 | A0A075B6K2 | 2356.6184 | 10233.6464 | 0.2037573 | 0.66969602 | 0.024618761 | 4.342513154 | 2.118530219 |
| STAU1 | O95793 | 634.9144 | 3003.5862 | 0.042261107 | 0.665914671 | 0.067889155 | 4.73069472 | 2.242052064 |
| ADH1A | P07327 | 914.1034 | 5399.4544 | 0.049492477 | 0.666478025 | 0.024618761 | 5.906831109 | 2.562384362 |
| LSM14A | Q8ND56 | 136.568 | 3488.0188 | 0.068886931 | 0.667407022 | 0.025347319 | 25.5405278 | 4.674716434 |
| FCGR3B | O75015 | 136.568 | 4043.1218 | 0.055706154 | 0.666845776 | 0.025347319 | 29.60519155 | 4.887778283 |
| NCF4 | Q15080 | 136.568 | 4734.1018 | 0.112645559 | 0.668330509 | 0.025347319 | 34.66479556 | 5.115399347 |
| CRTAP | O75718 | 3176.9844 | 119658.7204 | 0.191823414 | 0.669324939 | 0.024618761 | 37.6642455 | 5.235123724 |
