# Supplementary material for: Plasma proteome profiling identifies XPNPEP3 as a novel biomarker associated with metabolic dysfunction-associated steatotic liver disease in patients with type 2 diabetes mellitus
Source: Ann Med. 2026 Apr 13;58(1):2654911. doi: 10.1080/07853890.2026.2654911 (PMC13078656; doi:10.1080/07853890.2026.2654911)
Supplement: Table SIII Protein Protein Interaction Network.docx [file IANN_A_2654911_SM6031.docx]

**Table SIII** Protein-Protein Interaction (PPI) Network

| **Node1** | **Interaction** | **Node2** | **Source** |
| --- | --- | --- | --- |
| UQCRC1 | pp | NDUFS2 | experimental |
| UQCRC1 | pp | ATP5MG | textmining |
| UQCRC1 | pp | NDUFV2 | experimental |
| UQCRC1 | pp | ACADVL | textmining |
| UQCRC1 | pp | VDAC1 | textmining |
| UQCRC1 | pp | NDUFS1 | experimental |
| UQCRC1 | pp | COX6C | experimental |
| UQCRC1 | pp | SDHA | textmining |
| UQCRC1 | pp | SDHB | textmining |
| UQCRC1 | pp | UQCRC2 | experimental |
| UQCRC1 | pp | PHB2 | experimental |
| UQCRC1 | pp | UQCRQ | experimental |
| UQCRC1 | pp | NDUFA4 | textmining |
| UQCRC1 | pp | XPNPEP3 | experimental |
| ADH1A | pp | MAOB | database |
| ADH1A | pp | DECR1 | textmining |
| PSMD5 | pp | PSMD4 | textmining |
| SNAP29 | pp | B4GALT1 | database |
| SNAP29 | pp | STX4 | database |
| SNAP29 | pp | STON2 | textmining |
| NID2 | pp | NID1 | database |
| PYGB | pp | TXNDC5 | database |
| PYGB | pp | CYB5R3 | database |
| PYGB | pp | ACTR2 | database |
| CSNK2A1 | pp | MAPK14 | experimental |
| CSNK2A1 | pp | EEF1B2 | experimental |
| CSNK2A1 | pp | YWHAQ | experimental |
| DECR1 | pp | P4HB | textmining |
| DECR1 | pp | GOT2 | textmining |
| DECR1 | pp | MAOB | textmining |
| DECR1 | pp | DDAH2 | textmining |
| DECR1 | pp | VDAC1 | textmining |
| DECR1 | pp | MAPK14 | textmining |
| DECR1 | pp | CYB5R3 | textmining |
| DECR1 | pp | PRDX3 | textmining |
| DECR1 | pp | IMMT | textmining |
| DECR1 | pp | NCF4 | textmining |
| DECR1 | pp | MAP3K5 | textmining |
| DECR1 | pp | GLRX | textmining |
| DECR1 | pp | HSD17B12 | textmining |
| DECR1 | pp | EGF | textmining |
| DECR1 | pp | HADHB | textmining |
| DECR1 | pp | PRDX4 | textmining |
| DECR1 | pp | SHMT2 | textmining |
| SFXN3 | pp | UQCRQ | experimental |
| SLC25A11 | pp | HSD17B12 | textmining |
| SLC25A11 | pp | VDAC1 | experimental |
| SLC25A11 | pp | TUBB6 | textmining |
| USP5 | pp | PSMD4 | textmining |
| USP5 | pp | COPB1 | textmining |
| USP5 | pp | UBQLN1 | experimental |
| USP5 | pp | DIABLO | textmining |
| USP5 | pp | UBA7 | textmining |
| MAPK14 | pp | PRDX3 | textmining |
| MAPK14 | pp | NCF4 | database |
| MAPK14 | pp | LCK | database |
| MAPK14 | pp | MAP3K5 | database |
| MAPK14 | pp | EGF | textmining |
| MAPK14 | pp | NCK2 | database |
| MAPK14 | pp | DIABLO | textmining |
| NCK2 | pp | ARPC5 | database |
| NCK2 | pp | ARPC4 | database |
| NCK2 | pp | LCK | database |
| NCK2 | pp | ACTR2 | database |
| GOT2 | pp | MTTP | textmining |
| GOT2 | pp | SDHA | database |
| GOT2 | pp | CAD | database |
| GOT2 | pp | MPST | database |
| GOT2 | pp | SHMT2 | textmining |
| GOT2 | pp | SDHB | database |
| RTN2 | pp | VAPB | experimental |
| RTN2 | pp | RAB3GAP2 | textmining |
| COPB1 | pp | TMED7 | database |
| COPB1 | pp | TMED10 | database |
| COPB1 | pp | COPA | experimental |
| COPB1 | pp | CAD | experimental |
| COPB1 | pp | TGOLN2 | textmining |
| COPB1 | pp | HSD17B12 | experimental |
| COPB1 | pp | DYNLL1 | database |
| COPB1 | pp | AP1G1 | database |
| COPB1 | pp | DCTN1 | database |
| ACTN4 | pp | TRPC6 | textmining |
| ACTN4 | pp | PCYOX1L | database |
| ACTN4 | pp | PDLIM7 | experimental |
| ACTN4 | pp | ITGAV | database |
| ACTN4 | pp | EGF | database |
| ACTN4 | pp | ACTR2 | textmining |
| UGGT1 | pp | P4HB | textmining |
| UGGT1 | pp | DNAJB11 | textmining |
| UGGT1 | pp | ERAP1 | textmining |
| UGGT1 | pp | CALR | textmining |
| UGGT1 | pp | PDIA6 | textmining |
| UGGT1 | pp | GANAB | textmining |
| UGGT1 | pp | HYOU1 | textmining |
| UGGT1 | pp | PDIA4 | textmining |
| UGGT1 | pp | PDIA3 | textmining |
| LRPPRC | pp | MRPL12 | textmining |
| LRPPRC | pp | VDAC1 | textmining |
| LRPPRC | pp | PDHA1 | textmining |
| LRPPRC | pp | NDUFS1 | textmining |
| LRPPRC | pp | SDHA | textmining |
| ITGAV | pp | YWHAQ | experimental |
| ITGAV | pp | NID1 | experimental |
| ITGAV | pp | CALR | experimental |
| ITGAV | pp | LCK | database |
| ERP29 | pp | P4HB | textmining |
| ERP29 | pp | PDIA6 | textmining |
| ERP29 | pp | ARPC5L | textmining |
| ERP29 | pp | PDIA4 | textmining |
| ERP29 | pp | PDIA3 | textmining |
| ERP29 | pp | CALR | textmining |
| ERP29 | pp | TXNDC5 | textmining |
| NDST1 | pp | TM9SF2 | textmining |
| SLA2 | pp | LCK | database |
| NID1 | pp | MMP19 | database |
| SDHA | pp | NDUFS2 | experimental |
| SDHA | pp | ATP5MG | textmining |
| SDHA | pp | VDAC1 | textmining |
| SDHA | pp | CYB5R3 | experimental |
| SDHA | pp | NDUFS1 | textmining |
| SDHA | pp | UQCRC2 | textmining |
| SDHA | pp | GPD2 | textmining |
| SDHA | pp | NDUFV2 | textmining |
| SDHA | pp | ACADVL | textmining |
| SDHA | pp | PDHA1 | textmining |
| SDHA | pp | SHMT2 | textmining |
| SDHA | pp | SDHB | experimental |
| SDHA | pp | UQCRQ | textmining |
| EGF | pp | P4HB | textmining |
| EGF | pp | ARPC4 | database |
| EGF | pp | LRIG1 | database |
| EGF | pp | LCK | database |
| EGF | pp | PCYOX1L | database |
| EGF | pp | TGOLN2 | database |
| EGF | pp | ARPC5 | database |
| EGF | pp | DNM2 | database |
| EGF | pp | IGFBP1 | textmining |
| EGF | pp | STON2 | database |
| EGF | pp | ACTR2 | database |
| VDAC1 | pp | P4HB | textmining |
| VDAC1 | pp | CISD2 | experimental |
| VDAC1 | pp | CALR | textmining |
| VDAC1 | pp | ITPR1 | textmining |
| VDAC1 | pp | IMMT | textmining |
| VDAC1 | pp | DIABLO | textmining |
| VDAC1 | pp | SLC25A12 | database |
| VDAC1 | pp | SDHB | textmining |
| VDAC1 | pp | UQCRC2 | textmining |
| VDAC1 | pp | PHB2 | experimental |
| RHOF | pp | ACTR2 | textmining |
| UQCRC2 | pp | NDUFS2 | experimental |
| UQCRC2 | pp | ATP5MG | textmining |
| UQCRC2 | pp | NDUFV2 | experimental |
| UQCRC2 | pp | HADHB | experimental |
| UQCRC2 | pp | GANAB | experimental |
| UQCRC2 | pp | NDUFS1 | experimental |
| UQCRC2 | pp | IMMT | textmining |
| UQCRC2 | pp | COX6C | experimental |
| UQCRC2 | pp | SDHB | textmining |
| UQCRC2 | pp | NLRX1 | textmining |
| UQCRC2 | pp | UQCRQ | experimental |
| UQCRC2 | pp | NDUFA4 | database |
| LRIG1 | pp | RFTN1 | textmining |
| CISD2 | pp | ITPR1 | textmining |
| IGFBP1 | pp | P4HB | database |
| IGFBP1 | pp | PDIA6 | database |
| IGFBP1 | pp | TGOLN2 | database |
| HSD17B12 | pp | TMED7 | textmining |
| HSD17B12 | pp | HADHB | textmining |
| PDIA4 | pp | P4HB | experimental |
| PDIA4 | pp | DNAJB11 | textmining |
| PDIA4 | pp | PSMD4 | experimental |
| PDIA4 | pp | GANAB | textmining |
| PDIA4 | pp | CALR | textmining |
| PDIA4 | pp | PRDX4 | textmining |
| PDIA4 | pp | HYOU1 | textmining |
| ARPC5 | pp | CPVL | textmining |
| ARPC5 | pp | ARPC5L | database |
| ARPC5 | pp | DNM2 | database |
| ARPC5 | pp | STON2 | database |
| ARPC5 | pp | ACTR2 | experimental |
| ARPC5 | pp | TGOLN2 | database |
| ARPC5 | pp | ARPC4 | experimental |
| GUCY1A1 | pp | NME2 | database |
| GUCY1A1 | pp | DYNLL1 | textmining |
| ERAP1 | pp | XPNPEP3 | textmining |
| ERAP1 | pp | CALR | textmining |
| ERAP1 | pp | PDIA3 | textmining |
| DEFA4 | pp | ARMC8 | database |
| PRDX3 | pp | P4HB | textmining |
| PRDX3 | pp | TXNDC5 | experimental |
| PRDX3 | pp | GLRX | textmining |
| PRDX3 | pp | PDIA6 | textmining |
| PRDX3 | pp | PDIA3 | textmining |
| PDIA3 | pp | P4HB | experimental |
| PDIA3 | pp | DNAJB11 | textmining |
| PDIA3 | pp | GANAB | database |
| PDIA3 | pp | MTTP | textmining |
| PDIA3 | pp | GLRX | textmining |
| PDIA3 | pp | HLA-G | database |
| PDIA3 | pp | CALR | textmining |
| PDIA3 | pp | PRDX4 | textmining |
| PDIA3 | pp | GARS1 | experimental |
| PDIA3 | pp | HYOU1 | textmining |
| ATP5MG | pp | NDUFS2 | textmining |
| ATP5MG | pp | NDUFV2 | textmining |
| ATP5MG | pp | ACADVL | experimental |
| ATP5MG | pp | IMMT | textmining |
| ATP5MG | pp | AK2 | database |
| ATP5MG | pp | SDHB | textmining |
| ATP5MG | pp | UQCRQ | textmining |
| ATP5MG | pp | ATP6V1H | database |
| ATP5MG | pp | NDUFA4 | textmining |
| TMED10 | pp | TMED7 | database |
| TMED10 | pp | COPA | database |
| TMED10 | pp | UBQLN1 | textmining |
| TMED10 | pp | DYNLL1 | database |
| TMED10 | pp | HYOU1 | experimental |
| TMED10 | pp | SSR4 | textmining |
| TMED10 | pp | DCTN1 | database |
| ITPR1 | pp | TRPC6 | textmining |
| ITPR1 | pp | CALR | textmining |
| GPD2 | pp | SDHB | experimental |
| GPD2 | pp | UQCRQ | experimental |
| SSR4 | pp | VAPB | experimental |
| STX4 | pp | VAPB | experimental |
| STX4 | pp | CALR | database |
| TUBB6 | pp | PFDN2 | database |
| TUBB6 | pp | PFDN5 | database |
| CALR | pp | P4HB | textmining |
| CALR | pp | DNAJB11 | textmining |
| CALR | pp | TXNDC5 | textmining |
| CALR | pp | GANAB | experimental |
| CALR | pp | TGOLN2 | textmining |
| CALR | pp | HLA-G | database |
| CALR | pp | PDIA6 | textmining |
| CALR | pp | PRDX4 | textmining |
| CALR | pp | HYOU1 | textmining |
| IMPDH2 | pp | ENTPD5 | database |
| CRTAP | pp | P4HB | database |
| HADHB | pp | NME2 | textmining |
| HADHB | pp | ACADSB | database |
| HADHB | pp | ACADVL | database |
| HADHB | pp | SDHB | textmining |
| HADHB | pp | PCCB | database |
| NDUFV2 | pp | NDUFS2 | experimental |
| NDUFV2 | pp | NDUFS1 | experimental |
| NDUFV2 | pp | SDHB | experimental |
| NDUFV2 | pp | UQCRQ | experimental |
| NDUFV2 | pp | NDUFA4 | database |
| P4HB | pp | GPNMB | textmining |
| P4HB | pp | DNAJB11 | textmining |
| P4HB | pp | GANAB | experimental |
| P4HB | pp | MTTP | database |
| P4HB | pp | MAP3K5 | textmining |
| P4HB | pp | TGOLN2 | database |
| P4HB | pp | GLRX | textmining |
| P4HB | pp | PDIA6 | database |
| P4HB | pp | PRDX4 | textmining |
| P4HB | pp | UBQLN1 | experimental |
| P4HB | pp | HYOU1 | textmining |
| UBA7 | pp | UBQLN1 | experimental |
| SHMT2 | pp | GARS1 | textmining |
| MRPL12 | pp | AK2 | experimental |
| ENTPD5 | pp | NME2 | database |
| LCK | pp | GRAP2 | database |
| LCK | pp | YWHAQ | experimental |
| LCK | pp | DIABLO | textmining |
| NDUFA4 | pp | NDUFS2 | database |
| NDUFA4 | pp | NDUFS1 | database |
| NDUFA4 | pp | COX6C | database |
| NDUFA4 | pp | SDHB | textmining |
| NDUFA4 | pp | UQCRQ | database |
| GANAB | pp | TXNDC5 | textmining |
| GANAB | pp | HYOU1 | textmining |
| PEAR1 | pp | TMCC2 | textmining |
| ARPC5L | pp | ARPC4 | experimental |
| ARPC5L | pp | ACTR2 | experimental |
| AK2 | pp | NME2 | database |
| RAB3GAP2 | pp | DYNLL1 | database |
| RAB3GAP2 | pp | DCTN1 | database |
| MAP3K5 | pp | DYRK1A | experimental |
| MAP3K5 | pp | GLRX | textmining |
| ATP6V1H | pp | UBQLN1 | experimental |
| CYB5R3 | pp | TXNDC5 | database |
| CYB5R3 | pp | ACTR2 | database |
| DCTN1 | pp | TMED7 | database |
| DCTN1 | pp | COPA | database |
| DCTN1 | pp | VAPB | textmining |
| DCTN1 | pp | UBQLN1 | textmining |
| DCTN1 | pp | DYNLL1 | database |
| STUM | pp | GARS1 | textmining |
| NDUFS2 | pp | NDUFS1 | experimental |
| NDUFS2 | pp | SDHB | textmining |
| NDUFS2 | pp | UQCRQ | experimental |
| PFDN2 | pp | ACTBL2 | textmining |
| PFDN2 | pp | PFDN5 | experimental |
| COPA | pp | TMED7 | database |
| COPA | pp | DYNLL1 | database |
| ACADSB | pp | PCCB | textmining |
| PSMD4 | pp | PDIA6 | experimental |
| PSMD4 | pp | UBQLN1 | textmining |
| SDHB | pp | NDUFS1 | textmining |
| SDHB | pp | PDHA1 | experimental |
| SDHB | pp | UQCRQ | textmining |
| ACTR2 | pp | TXNDC5 | database |
| ACTR2 | pp | ARPC4 | experimental |
| ACTR2 | pp | TGOLN2 | database |
| ACTR2 | pp | DNM2 | database |
| ACTR2 | pp | STON2 | database |
| UQCRQ | pp | NDUFS1 | experimental |
| UQCRQ | pp | IMMT | experimental |
| UQCRQ | pp | COX6C | experimental |
| ACOT9 | pp | PRDX4 | textmining |
| PRDX4 | pp | TXNDC5 | experimental |
| PRDX4 | pp | GLRX | textmining |
| PRDX4 | pp | PDIA6 | experimental |
| TXNDC5 | pp | IMMT | textmining |
| TXNDC5 | pp | TGOLN2 | database |
| TXNDC5 | pp | DNM2 | database |
| TXNDC5 | pp | HYOU1 | textmining |
| TXNDC5 | pp | AP1G1 | database |
| PDHA1 | pp | NDUFS1 | textmining |
| ASAH1 | pp | ARMC8 | database |
| DNM2 | pp | ARPC4 | database |
| DNM2 | pp | TGOLN2 | database |
| DNM2 | pp | STON2 | database |
| DNM2 | pp | AP1G1 | database |
| GARS1 | pp | HARS1 | textmining |
| DYNLL1 | pp | TMED7 | database |
| AP1G1 | pp | TMED7 | database |
| AP1G1 | pp | TGOLN2 | database |
| MPST | pp | VAPB | experimental |
| MYCBP | pp | PFDN5 | textmining |
| DYRK1A | pp | SRSF5 | experimental |
| PDIA6 | pp | DNAJB11 | textmining |
| PDIA6 | pp | TGOLN2 | database |
| PDIA6 | pp | HYOU1 | textmining |
| TGOLN2 | pp | STON2 | database |
| TGOLN2 | pp | ARPC4 | database |
| IMMT | pp | SLC25A12 | experimental |
| IMMT | pp | PHB2 | textmining |
| ARPC4 | pp | STON2 | database |
| DNAJB11 | pp | HYOU1 | textmining |
| IVD | pp | PCCB | textmining |
| PCCB | pp | SRSF5 | textmining |
| ALYREF | pp | SRSF5 | database |
