# Supplementary figures and images for: Plasma proteome profiling identifies XPNPEP3 as a novel biomarker associated with metabolic dysfunction-associated steatotic liver disease in patients with type 2 diabetes mellitus
Source: Ann Med. 2026 Apr 13;58(1):2654911. doi: 10.1080/07853890.2026.2654911 (PMC13078656; doi:10.1080/07853890.2026.2654911)

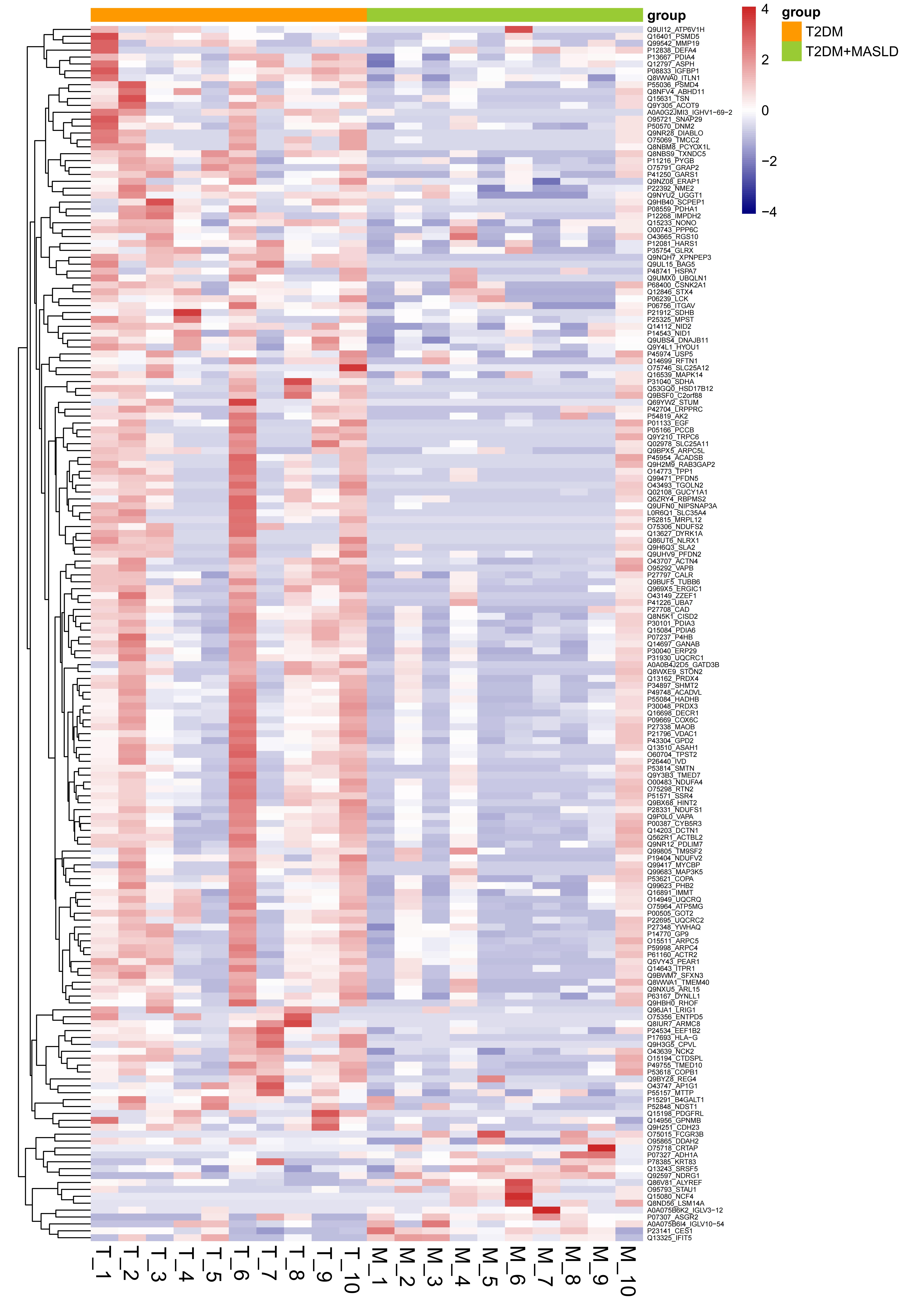

Supplement: FigureS1.jpg [file IANN_A_2654911_SM6030.jpg]
