# Supplementary material for: Plasma proteome profiling identifies XPNPEP3 as a novel biomarker associated with metabolic dysfunction-associated steatotic liver disease in patients with type 2 diabetes mellitus
Source: Ann Med. 2026 Apr 13;58(1):2654911. doi: 10.1080/07853890.2026.2654911 (PMC13078656; doi:10.1080/07853890.2026.2654911)
Supplement: Table SI Clinical Characteristics of 24 T2DM Patients With or Without MASLD for IHC.docx [file IANN_A_2654911_SM6029.docx]

| **Variables** | **T2DM(n=12)** | **T2DM+MASLD(n=12)** | **Effect Size (95% CI)** | ***P* value** |
| --- | --- | --- | --- | --- |
| Gender |  |  | 1.43 (0.26, 8.66) | 0.670 |
| Male | 9(75%) | 8(66.67%) |  |  |
| Female | 3(25%) | 4(33.33%) |  |  |
| Age (y) | 65.32±6.15 | 66.26±7.06 | -0.94 (-6.41, 3.73) | 0.623 |
| Diabetes duration (m) | 54.00(10.00, 120.00) | 42.00(15.00, 108.00) | -12.00 (-45.60, 21.60) | 0.482 |
| BMI (kg/m^2^) | 22.65±1.82 | 24.12±3.51 | 1.45 (-0.75, 3.65) | 0.185 |
| FPG (mmol/L) | 8.52±4.13 | 8.84±2.53 | 0.33 (-2.45, 3.11) | 0.812 |
| ALB (g/L) | 41.95±4.05 | 42.18±3.67 | 0.85 (-2.15, 3.85) | 0.565 |
| ALT (U/L) | 15.00(12.00, 18.50) | 44.00(32.00, 60.00) | 29.00(14.00, 44.00) | 0.003 |
| AST (U/L) | 19.00(14.00, 27.00) | 36.00(28.00, 50.00) | 17.00(5.00, 29.00) | 0.048 |
| ALP (U/L) | 80.00(64.00, 96.00) | 96.00(73.00, 118.00) | 16.00 (-10.00, 42.00) | 0.171 |
| GGT (U/L) | 23.00(15.00, 36.00) | 55.00(40.00, 85.00) | 32.00(14.00, 50.00) | 0.009 |
| TG (mmol/L) | 1.18±0.32 | 1.81±0.35 | 0.62 (0.35, 0.89) | 0.021 |
| TCH (mmol/L) | 4.63±0.68 | 4.98±0.81 | 0.35 (-0.25, 0.93) | 0.232 |
| Cre (μmol/L) | 68.61±14.82 | 63.38±8.53 | -5.31 (-15.21, 7.17) | 0.285 |
| Hb (g/L) | 132.40±15.36 | 140.93±13.54 | 8.46 (-6.25, 21.61) | 0.185 |

**Table SI** Clinical Characteristics of 24 T2DM Patients With or Without MASLD for IHC.

**Note:** Continuous variables conforming to a normal distribution are presented as mean ± SD and compared using the independent samples t-test, with the mean difference and its 95% CI reported. Variables not conforming to a normal distribution are presented as median (IQR) and compared using the Mann-Whitney U test, with the Hodges–Lehmann estimator of the median difference and its 95% CI reported. Categorical variables are presented as number (percentage) and compared using the chi-square test, with the odds ratio and its 95% CI reported.

**Abbreviations:** T2DM, Type 2 Diabetes Mellitus; MASLD, Metabolic Dysfunction-associated Steatotic Liver Disease; IHC, Immunohistochemistry; CI, confidence interval; BMI, Body Mass Index; FPG, Fasting Plasma Glucose; ALB, Albumin; ALT, Alanine Aminotransferase; AST, Aspartate Aminotransferase; ALP, Alkaline Phosphatase; GGT, Gamma-Glutamyl Transpeptidase; TG, Triglycerides; TCH, Total Cholesterol; Cre, Creatinine; Hb, Hemoglobin.
